# Culture-Independent Metagenomic Surveillance of Commercially Available Probiotics with High-Throughput Next-Generation Sequencing

**DOI:** 10.1128/mSphere.00057-16

**Published:** 2016-03-30

**Authors:** Jennifer N. Patro, Padmini Ramachandran, Tammy Barnaba, Mark K. Mammel, Jada L. Lewis, Christopher A. Elkins

**Affiliations:** Division of Molecular Biology, Center for Food Safety and Applied Nutrition, U.S. Food and Drug Administration, Laurel, Maryland, USA; University of Wisconsin, Madison

**Keywords:** Probiotics, dietary supplements, metagenomics, whole-genome sequencing, lactic acid bacteria

## Abstract

Millions of people consume dietary supplements either following a doctor’s recommendation or at their own discretion to improve their overall health and well-being. This is a rapidly growing trend, with an associated and expanding manufacturing industry to meet the demand for new health-related products. In this study, we examined the contents and microbial viability of several popular probiotic products on the United States market. Culture-independent methods are proving ideal for fast and efficient analysis of foodborne pathogens and their associated microbial communities but may also be relevant for analyzing probiotics containing mixed microbial constituents. These products were subjected to next-generation whole-genome sequencing and analyzed by a custom in-house-developed k-mer counting method to validate manufacturer label information. In addition, the batch variability of respective products was examined to determine if any changes in their formulations and/or the manufacturing process occurred. Overall, the products we tested adhered to the ingredient claims and lot-to-lot differences were minimal. However, there were a few discrepancies in the naming of closely related *Lactobacillus* and *Bifidobacterium* species, whereas one product contained an apparent *Enterococcus* contaminant in two of its three lots. With the microbial contents of the products identified, we used traditional PCR and colony counting methods to comparatively assess our results and verify the viability of the microbes in these products with regard to the labeling claims. Of all the supplements examined, only one was found to be inaccurate in viability. Our use of next-generation sequencing as an analytical tool clearly demonstrated its utility for quickly analyzing commercially available products containing multiple microbes to ensure consumer safety.

**IMPORTANCE** The rapidly growing supplement industry operates without a formal premarket approval process. Consumers rely on product labels to be accurate and true. Those products containing live microbials report both identity and viability on most product labels. This study used next-generation sequencing technology as an analytical tool in conjunction with classic culture methods to examine the validity of the labels on supplement products containing live microbials found in the United States marketplace. Our results show the importance of testing these products for identity, viability, and potential contaminants, as well as introduce a new culture-independent diagnostic approach for testing these products.

**Podcast**: A podcast concerning this article is available.

## Abstract (precis)

The rapidly growing supplement industry operates without a formal premarket approval process. Consumers rely on product labels to be accurate and true. Those products containing live microbials report both identity and viability on most product labels. This study used next-generation sequencing technology as an analytical tool in conjunction with classic culture methods to examine the validity of the labels on supplement products containing live microbials found in the United States marketplace. Our results show the importance of testing these products for identity, viability, and potential contaminants, as well as introduce a new culture-independent diagnostic approach for testing these products.

## INTRODUCTION

Probiotics are defined by the World Health Organization (WHO) as “live microorganisms which when administered in adequate amounts confer a health benefit on the host.” (1, 2) Additionally, it has been recommended recently by the International Association of Probiotics and Prebiotics that the term “probiotic” be used only for products that “deliver live microorganisms with a suitable viable count of well-defined strains with a reasonable expectation of delivering benefits for the well-being of the host” (3, 4). Microbes can occur naturally in foods, be intentionally added, or be packaged solely as a dietary supplement (5). The availability of these products to the consumer is reflected by the expansion of the dietary supplement business in the United States, which is now a $30 billion dollar/year global industry (6).

The majority of probiotic products contain *Lactobacillus*, *Bifidobacterium*, *Pediococcus*, *Streptococcus*, and *Lactococcus* species as either a mixture or single isolates. While these products report the number of CFU present per serving, as well as the type of microbes present in their product, on the label, they are not required to include this information. These types of products are packaged in a variety of ways, such as in capsule, gummy, or powder form. Probiotics commonly found on store shelves can be marketed to specific consumers, for example, specifically targeting women or children. The awareness that probiotics have the potential to affect an individual’s health has increased over the past several years. They function in modulating the host immune response, preventing the colonization of pathogenic organisms, and/or establishing a proper balance of the host gut microbiota (7–9). Recently, probiotic use has been extended beyond diet supplementation and has been used in clinical situations (7, 10). This alternative application as a medical option raises concerns about the safety of these products for human consumption and the possibility that they may require additional manufacturing process control. Therefore, an accurate description of the microbes present in these products, as well as determination of the presence of any microbial contaminants, specifically, those that may pose a health risk, is important for public safety and the proper labeling of these products.

Errors in labeling accuracy due to the miscalculation of levels of bacteria or the proper taxonomic identification of bacterial species have been reported extensively worldwide (11–17). However, most of these studies used PCR or selective culture techniques, which are *a priori* limited to targeting certain bacteria in a mixed product. Moreover, such methods are labor intensive and may take many days to complete. On the contrary, species-agnostic approaches better capable of capturing unknown potential contaminants or mislabeled components would be optimal, especially if contained in a single platform. Genomic-scale taxonomy has become a popular identification technique, with the cost and speed of whole-genome sequencing rapidly decreasing over the past several years (18, 19). This technique can be used to easily identify species present in dietary supplements and determine any mislabeling of these products rapidly and accurately (20). In addition, whole-genome sequencing analysis of probiotics can determine the presence of any microbial contaminant that can have adverse effects on public health.

In this study, we used a culture-independent method involving shotgun next-generation sequencing to determine the contents of various types of commercially available probiotics. This approach will provide an analytical laboratory the ability to determine, in a single assay, the types and relative amounts of bacteria present, as well as the presence of bacteria not reported on the label. The existence of a microbial contaminant found during sequencing can be confirmed by PCR with strain-specific primers and/or selective media. In addition to the identities of all microbes, the viability of these probiotic organisms is also of concern. Labeling claims commonly include the amount of live microorganisms per capsule. Since sequencing cannot determine whether an organism that is present in a product is viable, the accuracy of these claims was verified by using standard culturing techniques and specific media to isolate and determine the viability of all of the microbes in each product.

## RESULTS

### Sequencing quantitation and limit of detection.

In order to determine whether the DNA extraction technique used would yield the expected quantitative results, two different mixed mock samples containing various amounts of representative species were developed (Table 1). In the first method, single-colony isolates of known strains were grown in their respective media overnight and combined in known relative amounts. DNA was extracted from this mixture in the same manner as DNA was extracted from the probiotic products. In the second method, DNA was isolated from each single-colony isolate and then combined into one sample of known relative amounts. With both methods, we comparatively assessed sequencing read quantitation against these artificially constructed metagenomic samples.

**TABLE 1:** Percent species and DNA abundances determined by k-mer analysis of mixed mock sample sequences

Species	% Abundance	
	Read	Mock
Mixed mock DNA	**	**
* Lactobacillus acidophilus*	41.6	45
* Lactobacillus plantarum*	35.5	35
* Lactobacillus zeae*	7.1	7
* Lactobacillus helveticus*	10.1	6
* Lactobacillus brevis*	4.1	3
*Lactobacillus casei* group	0.0	3
* Lactobacillus reuteri*	1.5	1
Mixed mock culture	**	**
* Lactobacillus acidophilus*	65.1	45
* Lactobacillus plantarum*	15.8	35
* Lactobacillus zeae*	10.5	7
* Lactobacillus helveticus*	4.1	6
* Lactobacillus brevis*	0.0	3
*Lactobacillus casei* group	4.1	3
* Lactobacillus reuteri*	0.0	1

**FIGURE 1:**
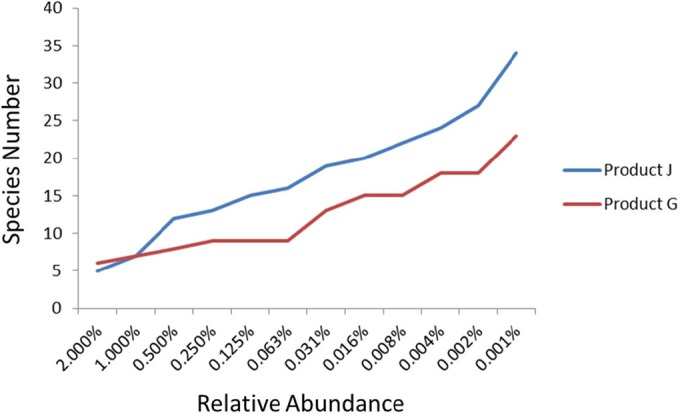
Bacterial species identified at <2% relative abundance in products G and J by k-mer analysis.

The relative amount of DNA obtained by each approach was determined by k-mer analysis. The mock culture mixture was not as accurate as the mock DNA mixture, which was extremely accurate for strains with abundances of >3% (Table 1). Below this level, the relative amount of each strain in both constructions was identified with less accuracy. While the mock culture mixture is less accurate quantitatively, the dominant species were properly identified and all species present at >3% were detected. Thus, sequencing has the qualitative ability to determine the dominant species present in the sample, although quantitation may be dependent on additional undefined factors such as relative lysis efficiency or inherent DNA stabilities in mixed culture lysis. We extended these findings with arguably the more complex products used in this study, one of which (product G) was extensively characterized for other purposes. Thus, preliminary k-mer sequence analysis of products G and J revealed a sharp increase in species identifications below approximately 1.5% relative sequence abundance (Fig. 1). For the purposes of our study, results below this level were considered noise and were scored appropriately (as not present in the sample). In addition, this was concluded because of the lack of false positives produced by organisms present at <1.5% throughout the rest of the study.

It is important in product surveillance to capture all of the organisms present in the samples without introducing false positives. Therefore, the cutoff of species present in a sample was established as 1.5%, with the caveat that quantitation of the relative abundances of the DNA species present cannot be established below 3%. We also examined the limit of detection of a strain particularly relevant to this study in a mixed sample. This was achieved by spiking 50 × 10^9^ CFU of probiotic product G with a known number of *Enterococcus faecium* cells (see Table S4 in the supplemental material). The relative abundance of *E. faecium* was determined by the in-house k-mer method described, and the results show that the contaminant is detectable when it is used to spike a sample at levels as low as 1.5%. With the exception of the lowest concentration of *E. faecium*, the relative amount was determined to be higher than the actual amount used to spike the product. However, the amount of *E. faecium* did increase at increasingly higher spiking levels. In addition, for other organisms in the product, relative sequencing yields tended to be consistent (demonstrating precision) across multiple repetitive samples.

### Metagenomic sequencing of probiotic products.

**FIGURE 2:**
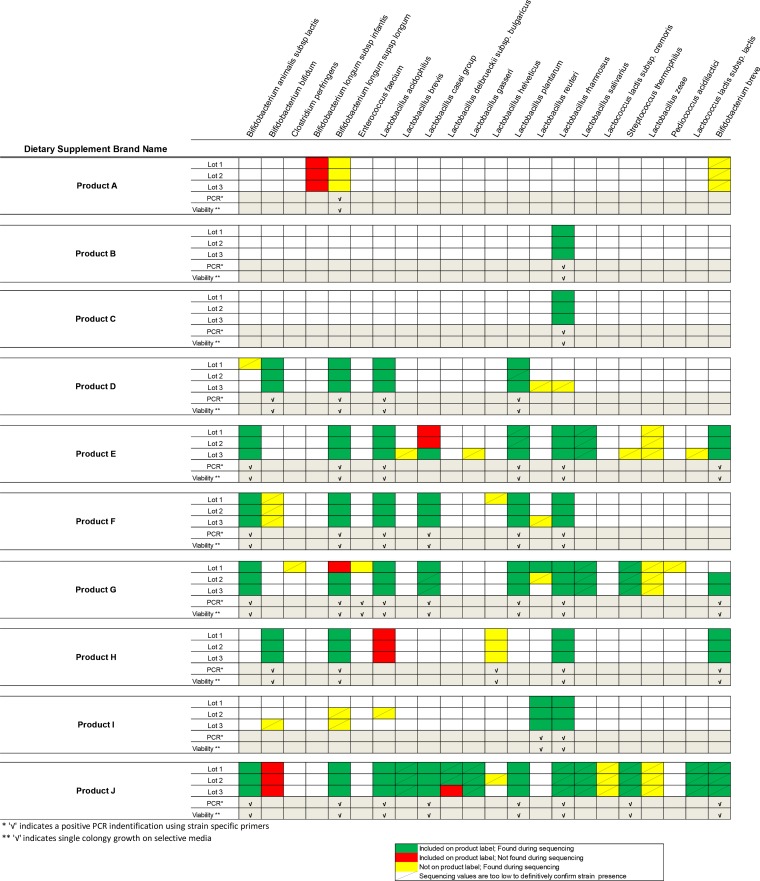
Summary qualitative analysis of metagenomic sequencing of various probiotic products and associated inconsistences with labeling.

**FIGURE 3:**
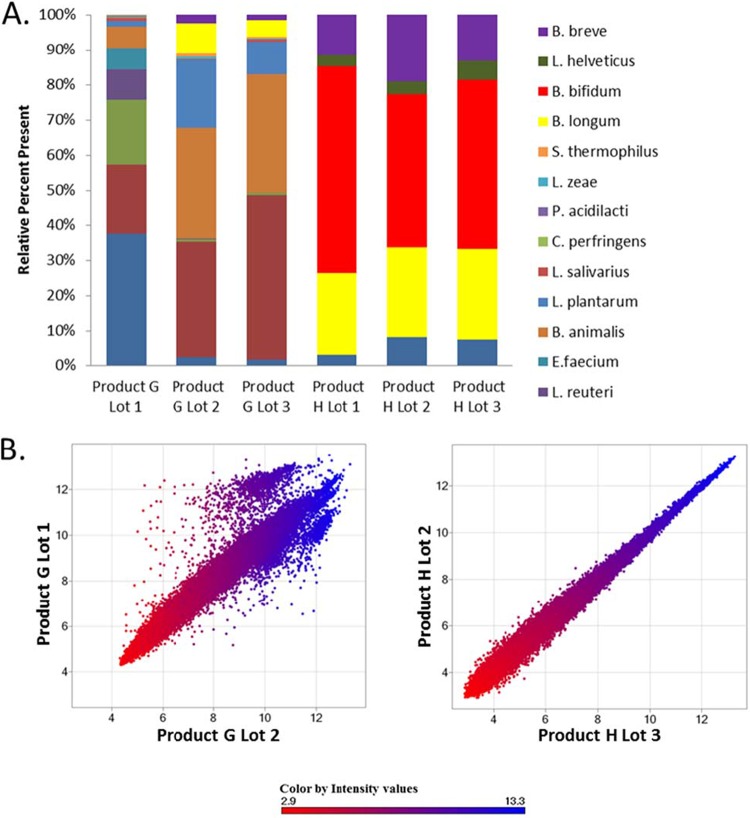
Batch variation of formulations from two products by two different genomic-scale techniques. (A) The relative abundance of each bacterium in a product as determined by k-mer analysis of metagenomic sequencing. The results are for two examples, products G and H, across three respective lots. (B) Analysis of the same samples with the custom-designed high-density GutProbe microarray platform (9). Each spot is a summarized probe set intensity (red shading, <8.5 absent; blue shading, ≥8.5 present) for a specific gene array from available whole genomes of human gut-associated species, including common probiotics. As has been shown previously, this genomic-scale genotyping is exquisitely sensitive for the determination of the identities and formulations of products, especially when they are assessed in pairwise comparisons.

Ten of the top-selling online probiotic products were tested for content with Illumina MiSeq. These products were assigned letters A to J. The reported ingredients of each product are listed in Table 2. The majority of these products have more than one microbe listed as ingredients on their labels and identify the microbes present in their product to the species level. Therefore, the ability to identify multiple types of bacteria without culturing makes this an ideal technique for identifying these products in comparison to the product labeling. However, this method could be used to identify supplement contents down to the strain level if this information is supplied by the manufacturer. We present a simplified version of the results in Fig. 2, showing bacteria (i) present on the label and detected via sequencing (green), (ii) present on the label but not detected via sequencing (red), and (iii) not on the label but detected during sequencing (yellow). In addition, some bacteria were also indicated that are below the limit of detection (detected at a relative abundance of 0.1 to 1.5%) but could be present in the sample. The relative abundance cutoff for a present call is 1.5%, that for a marginal call (represented by a line) is 0.1 to 1.5%, and that for an absent call is <0.1%. For two example products (G and H), the relative amounts of the microbes present are stratified and displayed across different product lots (Fig. 3A), as was done for all of the products (see Table S5 in the supplemental material). Of the 10 products examined, 1 (product G) potentially contained additional bacteria that were not indicated on the label, including a possible *E. faecium* contaminant (product G, lot 1; Fig. 2). This finding was further confirmed by culture methods and whole-genome sequencing (see below). In parallel, many constituent bacteria were assessed by PCR assay and viability testing. Thus, they were detectable with species-specific PCR primers (see Table S3 in the supplemental material) and were also grown on appropriate solid media to confirm their presence (see Tables S6 and S7 in the supplemental material).

**TABLE 2:** Comparison of product labeling values for viability and identity versus bacteriological culturing conducted in this study

Product	Ingredient(s)^a^	CFU count^b^
A	Bifantis contains 1 × 10^9^ CFU of *B. infantis* 35624 (4.0 mg) when manufactured and provides an effective level of bacteria (1 × 10^7^ CFU) until at least the “best-by” date	1 × 10^9^
B	10 × 10^9^ cells of *Lactobacillus* sp. strain GG, 10 × 10^9^ cells of inulin (chicory root extract) (200 mg)	10 × 10^9^
C	5 × 10^9^ cells of *Lactobacillus* sp. strain GG	5 × 10^9^
D	4 × 10^9^ cells of *L. acidophilus*, *B. bifidum*, *B. longum*, and *L. plantarum* OM	4 × 10^9^
E	12 × 10^9^ CFU of *L. acidophilus* la-14, 12 × 10^9^ CFU of *B. lactis* BI-04, 1 × 10^9^ CFU of *L. casei* Lc-11, 1 × 10^9^ CFU of *B. breve* Bb-03, 1 × 10^9^ CFU of *L. salivarius* Ls-33), 1 × 10^9^ CFU of *L. plantarum* Lp-115, 1 × 10^9^ CFU of *B. longum* Bl-05, 1 × 10^9^ CFU of *L. rhamnosus* Lr-32	30 × 10^9^
F	3.4 × 10^9^ CFU of *B. longum* BB536, *L. acidophilus* la-14, *B. lactis* BI-04, *L. rhamnosus* R0011, *L. casei* R0215, and *L. plantarum* R1012	3 × 10^9^
G^c^	Proprietary blend of 50 × 10^9^ cells of *L. paracasei*, *B. lactis*, *L. plantarum*, *L. acidophilus*, *B. longum*, *L. rhamnosus*, *S. thermophilus*, *L. casei*, *L. salivarius*, and *L. reuteri* strains	50 × 10^9^
H	100 × 10^9^ CFU of *B. breve* 129, *B. longum* 135, *B. bifidum* 132, *L. acidophilus* 122, and *L. rhamnosus* 111	80 × 10^9^
I	2.5 × 10^9^ CFU of *L. rhamnosus* GR-1, 2.5 × 10^9^ CFU of *L. reuteri* RC-14	5 × 10^9^
J	32 × 10^9^ cells of *B. bifidum*, *B. breve*, *B. lactis* (*infantis*), *B. lactis* HN019, *B. longum*, *L. acidophilus*, *L. brevis*, *L. bulgaricus*, *L. casei*, *L. gasseri*, *L. paracasei*, *L. plantarum*, *L. rhamnosus*, *L. salivarius*, *L. lactis*, and *S. thermophilus*	30 × 10^9^

^a^ As reported/labeled on product.

^b^ Experimentally determined in this study.

^c^ Product G contained a change in the formulation on the label in the lots tested in this study. Lot 1 is displayed here. In lots 2 and 3, *L. reuteri* was replaced with *B. breve*.

Sequencing analysis of these products determined several mislabeling issues, although many of these were simply apparent errors in nomenclature, i.e., *Lactobacillus acidophilus* as *L. helveticus* and *L. casei* as *L. zeae* (21–23). In addition, the label of one product (J) misidentified a common *Bifidobacterium* species as *Bifidobacterium lactis* (*infantis*) instead of *B. longum* subsp. *infantis* (Fig. 2) and revealed how frequent and obvious the mislabeling of these products has become. Our results show that this is a common practice; however, these organisms have not raised safety concerns in the past.

### Enumeration.

In addition to ingredient labeling, most probiotic products contain active-culture information. To test claim validity, each product was directly plated onto selective media for each organism identified during sequencing. The types of media used to examine the presence of each bacterium (see Tables S6 and S7 in the supplemental material) not only allowed us to determine the number of CFU in each product but also confirmed our sequencing results. All of the products contained the amounts of live cultures reported on their labels within 90%, except one product, H (Table 2), which was reported to contain 100 × 10^9^ CFU but contained only 80 × 10^9^ CFU.

### Confirmation of sequencing results.

The sequencing results revealed some labeling discrepancies that were further explored by other techniques. Two of the products (G and H) were examined with our custom-designed Affymetrix microarray, GutProbe (Fig. 3B). From our sequencing analysis, product H was qualitatively consistent across product lots, with only limited apparent quantitative differences (Fig. 3A), whereas product G had different product formulations in lots 1 and 2 (Table 2, footnote *c*). These observations were readily apparent with the GutProbe platform. Thus, pairwise scatterplot comparisons of product lot formulations that are similar should display equal hybridization intensities across the microarray, resulting in probe sets clustering tightly along the diagonal, as shown for product H (lots 2 and 3). However, formulation differences, such as those in product G, display biases in hybridization intensities specific to a respective lot (Fig. 3B).

In addition, all products were plated onto species-specific media in order to determine the morphological presence of bacteria detected via sequencing. All species, in general, were distinguished from one another on the basis of colony morphology with recorded colony descriptions (see Table S6 in the supplemental material). However, the colony morphologies of *L. salivarius*, *L. fermentum*, *L. casei*, and *L. paracasei* were not discernible in relation to one another. Therefore, we were unable to identify those organisms in two of the products (product G and J) on the basis of colony morphology. Custom-designed primers were used to confirm the diversity of the single-colony isolates (see Table S3 in the supplemental material). These additional methods are very important in confirming the presence of a contaminant like the one found in lot 1 of product G. It was determined via metagenomic sequencing that this sample contained a large relative amount of *E. faecium* that was not detected in the other two lots. As expected, we obtained a single-colony isolate from lot 1 on *Enterococcus* medium that was subsequently sequenced. We confirmed that the contaminant was *E. faecium*, with 80% of the reads mapping to the reference strain of *E. faecium*. Unlike the metagenomic results, which only identified *E. faecium* in lot 1, culturing of the two additional lots revealed the growth of *E. faecium* in lot 2. Indeed, a second isolate was obtained from lot 2 of product G and the genome sequences of the isolates from both lots were identical to one another. However, they did not match available reference strains in GenBank. We further assessed the sequences for virulence factors (24) against the virulence gene database MvirDB (http://mvirdb.llnl.gov/) but failed to identify any matches.

## DISCUSSION

The WHO defines probiotics as “live microorganisms which when administered in adequate amounts confer a health benefit on the host.” In today’s market, many food products are available with the intentional addition of live microbes with advertised generalized health benefits. Ten different probiotic supplements marketed in the United States were examined to verify the information presented on the label. A method to rapidly verify the labeling of these products, as well as identify any contaminants, would be useful for quality control and safety. Here, next-generation sequencing was used to meet these needs. Using this method would allow the testing of multiple products in one assay (up to 10, depending on the number of strains identified in each product), with results within 1 week. While this method is still costly, the expense associated with whole-genome sequencing technologies has been significantly declining over the past decade and continues to fall with the advent of next-generation platforms (18). This method’s comprehensive nature makes it worth the initial investment, as long as relative ease of postrun analysis continues to improve. In addition, since the labeling information on these products usually does not contain strain information or quantitative descriptions of each microbial constituent, this method could be useful in obtaining additional information beyond a given product label.

Most of these products were in capsule form, and DNA was directly isolated from the powder contained in the capsules; however, two of the products were in powder form. Regardless, since DNA isolation from all products started with a powder form, only one DNA isolation technique was used in this study. To establish this method as a viable way to determine relative amounts of bacteria, we used a mock sample with known relative amounts of bacteria. The results reveal that while each component of the sample is identified, the relative amounts vary from the known amounts, especially when the bacteria are present at <3%. On the basis of the mock sample analyses, a cutoff of 1.5% was established for the relative amount of bacteria in a sample. While the results are not accurate at relative abundances of <3%, the cutoff was chosen because of the lack of false positives present at levels of >1.5% (Table 1; Fig. 1). In addition, the limit of detection was further established by spiking a product sample with a known quantity of *E. faecium*. This organism is detected at levels as low as 1.5%. When dietary supplements were plated to obtain the total colony counts, we isolated and identified single colonies on the basis of their morphologies and confirmed their identities by PCR assay. The single-colony isolates were obtained only for organisms where the sequencing reads were >1.5%. This additional effort to confirm identities to the species level complemented the sequencing data to avoid any false-positive calls. We realize that these conclusions are based on a small data set, and they are intended as a first proof of principle to assess the potential of this technique for the identification of probiotic bacteria to the species level. We also realize that relative lysis methods and efficiencies need to be further determined given the recalcitrant nature of certain Gram-positive organisms.

Next-generation DNA sequencing has rapidly become a useful method to identify the contents of mixed samples (9, 25–28) and is quite applicable to the identification of all of the live-microbe ingredients in dietary supplement samples in this study. The volume of data generated by whole-genome sequencing was analyzed through a custom in-house pipeline. Our k-mer method was used to rapidly and effectively identify all of the ingredients in various dietary supplement samples to the species level. This approach can be tailored to the desired taxonomic resolution required, including the identity of an organism to the strain level. However, since most product labels do not contain this information, our results are reported to the species level to correspond to the product labeling information. Furthermore, this analysis method is able to determine the presence of specific contaminants in these products. Along these lines, the genomes included in the database are those of bacteria commonly found in the gut or typically found in marketed products. Thus, the database does not include common food contaminants but can be easily modified to include a wider variety of bacteria to test products more thoroughly for contamination.

Overall, high-throughput sequencing data revealed that half of the products analyzed matched the contents listed on their respective labels. Of note, these samples (products A to D and H) contained the least bacteria; all had five or fewer bacterial genera listed. Product A lists *B. longum* subsp. *infantis* on its label, and sequencing results revealed *B. longum* subsp. *longum*. These two *B. longum* subspecies are difficult to differentiate taxonomically, and this can easily lead to misidentification (29). In addition, primers were designed to distinguish between these two subspecies by PCR-based methods. However, product A was positive for both subspecies (*infantis* and *longum*) via PCR, indicating that whole-genome sequencing is the best method for the identification of this product’s contents. Furthermore, sequence analyses highlighted other common nomenclature errors (i.e., *L. acidophilus* and *L. helveticus*; *L. casei* and *L. zeae*) found in multiple products (E, H, and J) (9, 30). However, these mislabeling issues are less of a concern than others found in the products tested. *B. bifidum* was not detected in any of the three lots of product J tested (Fig. 2). Finally, it was apparent that manufacturers change their formulations from lot to lot, as shown in product G (Fig. 3A and B).

While this method is extremely useful as a screening tool to determine if any microbial contaminants are present in products, it should not be used as the sole method to screen live microbial products for contaminants. For example, sequencing showed that lot 1 of product G contained an *E. faecium* contaminant that was not detected in either lot 2 or 3. To further explore this result and confirm the presence of this organism, classical culture techniques were used with all three lots. When applied to *E. faecium*-specific media, both lots 1 and 2 produced growth. The growth obtained with lot 1 one was 3-fold greater than that obtained with lot 2 (data not shown), potentially revealing the reason why *E. faecium* was not detected by sequencing in lot 2. However, it is important to note that the sequencing approach did lead to further investigation of all of the lots of the product to confirm the presence of this bacterium.

The numbers of live cultures reported on product labels were tested by traditional culture-based techniques to verify labeling claims. While DNA sequencing can determine whether signatures of a specific organism are present, it may not determine viability by our approach; however, RNA profiling may have future utility in this regard. Therefore, it is necessary to use basic culture techniques as a secondary verification tool for sequencing approaches. All of the labels tested, except for that of product H, were accurate. This method is still extremely labor intensive and requires multiple media to cover all of the bacteria present in each product. Each product was stored at 4°C for no more than 8 months prior to testing. It would be of interest to determine the shelf lives of these products at various storage temperatures.

The methods currently used to identify microbes within dietary supplements are laborious and do not provide immediate identification to the species level when dealing with a mixed microbial community. Whole-genome sequencing offers an efficient, culture-independent method for quickly analyzing a complex sample. It can accurately identify all of the labeled ingredients, inclusive of any potential pathogens or contaminants, given appropriate depth and breadth of the associated bioinformatic database. However, at this point, it is not unilaterally capable of accurately identifying all of the microbes in a sample. In addition, whole-genome sequencing cannot yet establish the viability of the bacteria in a sample. Thus, culture techniques must be used in combination with this cutting-edge technique to obtain a more complete snapshot of the product. However, as we have demonstrated here in this proof of principle, analytical sequencing is extremely promising as a first step for screening product contents for contaminants and/or mislabeling and could be potentially applied to conventional foods.

## MATERIALS AND METHODS

### DNA isolation.

Samples used for validation were grown in their respective broths as recommended by the culture collection from which they were purchased. Once grown anaerobically for 24 to 36 h, the culture was pelleted (5,000 × *g*, 10 min) and resuspended in 750 µl of the lysis buffer included in the Qiagen DNeasy kit (Qiagen, Valencia, CA). DNA was isolated from probiotic samples (by breaking open capsules or direct isolation from powder forms) or the mock culture mixture with the Mo Bio PowerSoil DNA isolation mega-prep kit (Mo Bio, Carlsbad, CA) in accordance with the manufacturer’s instructions. This kit was used because of its ability to process a large volume of sample at once, which is needed to prevent clogging of the filters with capsule powder contents. DNA was extracted in accordance with the manufacturer’s protocol. DNA was then precipitated with 85 µM NaCl in cold 100% ethanol and spun (10,000 × *g*) for 30 min before the supernatant was discarded. Resuspended DNA (in 200 µl of water) was used for microarray analysis or sequencing. Three capsules were used in each preparation to obtain approximately 20 µg of DNA.

### Sequencing.

DNA libraries were generated with the Nextera kit (Illumina, San Diego, CA) in accordance with the manufacturer’s instructions. Up to three samples were multiplexed per run, depending on sample complexity, with Nextera index primers in accordance with the manufacturer’s protocol and then sequenced with the Illumina MiSeq 300- or 500-cycle cartridge kit.

### Sequence analysis.

All reads were trimmed with DynamicTrim v.1.13 prior to analysis (31). Metagenomic sequencing data sets were analyzed by using an in-house-developed k-mer database. The general approach to the development of the database has been described previously (32). Briefly, a comprehensive collection of the genome sequences of commercialized species present in the normal gut environment was downloaded from the NCBI database (see Table S2 in the supplemental material). The sequences were segmented into 25-bp (25-mer) nonoverlapping reads. K-mers not found in at least 2/3 of a set of additional genome sequences of the same species were removed along with any k-mer found in the genomes of other species. Typically, we retained 40,000 to 80,000 25-mers per species. The coverage of the 25-mers within the genome of each species was determined by testing each possible nonoverlapping 100-bp *in silico* read from the genome against the 25-mers and tallying the number of reads in the genome that were matched by the 25-mers.

For the analysis regimen *per se*, all 25-mers from each species database were placed into a trie structure. Each read from a metagenomic sequencing run was compared to the 25-mers in the trie. A read in which the majority of the 25-mer matches belonged to one species was identified as that species. The tally of matches from the sequencing run for each species was normalized by dividing the count by the number of *in silico* matched reads. The k-mer algorithm compares trimmed metagenomic reads against these short, specific 25-mers by using nucleotide BLAST searches in order to provide abundances for one or more sequenced metagenomes. This reduces the analysis time compared to that required for a BLAST search of the full catalog of microbial genomes, primarily because of the reduced size (25-mers) (33).

### Microarray hybridization and data analysis.

The FDA GutProbe array is a custom Affymetrix DNA microarray platform that contains the entire gene content derived from 10 genera, represented by 92 whole genomes and 229 plasmids. The DNA extracted from certain products in this study was subjected to digestion, labeling, hybridization, and scanning as previously described (9). The data were processed in R with the associated Affymetrix analysis package, and the visualization of scatter plots was performed as described previously (9).

### Selective culturing.

All products were plated on the following media for the growth of labeled ingredients: Luria-Bertani medium for the detection of any Gram-negative contaminants, complete supplement mixture for yeast detection, and potato dextrose agar for the detection of any mold, etc. The labeled serving size of each supplement was dissolved in 1 ml of water and then serially diluted 1:10 in MRS broth. Three separate dilutions per medium type were plated with 100 µl of the sample. Colonies were counted with a colony counter, and colonies were picked and stained by the Gram staining method.

### Culture enumeration.

Quantification of the bacteria in a given sample is routinely achieved by determining the total number of CFU grown on solid medium from serial dilutions and expressed as the number of CFU per gram or milliliter of the original sample. All of the plates were counted with a colony counter (Advanced Instruments, Norwood, MA). The bacteriological analytical manual recommends counting the colonies from the dilution that gives plate counts closest to 350 CFU and then estimating the total number of colonies (34). This number is then used as the estimated aerobic count. The lower limit of enumeration can be based on the limit of detection (25 CFU, from a countable range of 25 to 350 CFU).

### PCR detection and confirmation.

All single-colony isolates were incubated in 5 ml of MRS broth at 37°C under anaerobic conditions for 48 h. DNA was isolated from each sample by the DNeasy (Qiagen) extraction protocol for Gram-positive bacteria. The DNA was cleaned and concentrated with the Zymoclean DNA Clean & Concentrator kit (Zymo Research, Irvine, CA). The PCR assay for strain identification was performed with the Bio-Rad C1000 Touch Thermal Cycler (Bio-Rad, Hercules, CA). For each reaction, 1.5 µl of DNA was added to 1 µl of the forward primer, 1 µl of the reverse primer, 2 µl of the 5× Omni Klentaq PCR kit, and 5.5 µl of PCR Enhancer Cocktail 1 (DNA Polymerase Technology, St. Louis, MO). Strain-specific primers (see Table S3 in the supplemental material) were checked for specificity by using eight control samples: *Lactobacillus rhamnosus* ATCC 53103, *Lactobacillus delbrueckii* subsp*. bulgaricus* ATCC 11842 (ATCC, Manassas, VA), *Lactobacillus casei* subsp. *casei* DSM 20080, *Lactobacillus reuteri* ATCC 55730, *Lactobacillus plantarum* 14431, and *Lactobacillus casei* Lc-10. The primers were then used with single-colony DNA previously isolated from probiotic plates. The PCR conditions were as follows for all primers except for *fusA*: initial denaturation at 95°C for 2 min; 30 cycles of 94°C for 30 s, 65°C for 30 s, and 72°C for 30 s; and a final extension at 72°C for 5 min. For the *fusA* primer set, temperature for the 30-s extension was decreased to 63°C. All PCR products were analyzed by 1% agarose gel electrophoresis.

### Limits of detection assays. (i) DNA assay.

A DNA mock culture was prepared by mixing the DNA isolated from *L. acidophilus*, *L. plantarum*, *L. zeae*, *L. helveticus*, *L. brevis*, *L. casei*, and *L. reuteri*. The concentration of DNA was measured with the NanoDrop 2.1 (Thermo Scientific, Wilmington, DE). DNA was diluted to the same concentration across the samples and then mixed in various proportions (Table 1). The mixture was then subjected to the Nextera library prep protocol, and whole-genome sequencing was performed with MiSeq (Illumina).

### (ii) Culture assay.

*L. acidophilus*, *L. plantarum*, *L. zeae*, *L. helveticus*, *L. casei*, *L. reuteri*, *L. rhamnosus*, and *Streptococcus thermophilus* were all grown separately in MRS and incubated for 48 h at 37°C under anaerobic conditions. The cultures were all diluted with sterile 1:10 MRS medium, and optical density at 560 nm (OD_560_) was measured with a SpectraMax Spectrophotometer (Molecular Devices, Sunnyvale, CA). The cultures were diluted to achieve an OD_560_ of ~0.8 and mixed in various proportions (Table 1). The DNA was extracted from this culture mixture with the PowerSoil DNA extraction kit in accordance with the manufacturer’s instructions (Mo Bio). The DNA was subjected to Illumina sequencing as detailed above.

### Detection of *E. faecium* in product G and isolation.

The sequenced product containing signatures for *E. faecium* was plated on *Enterococcus* medium (Fisher Scientific, Frederick, MD). Agar plates were incubated at 37°C aerobically overnight. The single-colony isolates of *E. faecium* were incubated in *Enterococcus* broth (Fisher Scientific, Pittsburgh, PA) at 37°C aerobically overnight. DNA was isolated by the Qiagen DNA extraction protocol for Gram-positive bacteria. DNAs extracted from single-colony isolates of *E. faecium* were sequenced with Illumina MiSeq following preparation of the Nextera library. These sequences were compared to the reference strain by using seven multilocus sequence type identifiers.

### Level of *E. faecium* detected in a noncontaminated lot of product G.

*E. faecium* NCTC 7171 was grown overnight at 37°C aerobically in brain heart infusion medium. Different loads of cells (*E. faecium* NCTC 7171) were used to spike the product (see Table S4 in the supplemental material). The DNA was extracted with the Mo Bio PowerSoil extraction kit in accordance with the manufacturer’s suggested protocol. The extracted DNA was subjected to Nextera library preparation in accordance with the manufacturer’s instructions. Shotgun sequencing was performed with Illumina MiSeq (Illumina).

### Nucleotide sequence accession numbers.

Sequences were uploaded to the NCBI BioProject site under ID no. PRJNA291686; for the accession numbers assigned, see Table S1 in the supplemental material. The sequences obtained by Illumina MiSeq from single-colony isolates of *E. faecium* from product G were submitted to the GenBank Sequence Read Archive (for the accession numbers assigned, see Table S1 in the supplemental material).

10.1128/mSphere.00057-16.1 TABLE S1  NCBI Sequence Read Archive accession numbers and sample information from this study. Download TABLE S1, DOCX file, 0.01 MB. Copyright © 2016 Patro et al. 2016 Patro et al. This content is distributed under the terms of the Creative Commons Attribution 4.0 International license.

10.1128/mSphere.00057-16.2 TABLE S2  Composition and construction of k-mer database. Download TABLE S2, DOCX file, 0.1 MB. Copyright © 2016 Patro et al. 2016 Patro et al. This content is distributed under the terms of the Creative Commons Attribution 4.0 International license.

10.1128/mSphere.00057-16.3 TABLE S3  Custom primers designed to identify particular *Bifidobacterium*, *Lactobacillus*, and *Streptococcus* species by using unique housekeeping genes in each species. Download TABLE S3, DOCX file, 0.01 MB. Copyright © 2016 Patro et al. 2016 Patro et al. This content is distributed under the terms of the Creative Commons Attribution 4.0 International license.

10.1128/mSphere.00057-16.4 TABLE S4  Metagenomic sequencing-based detection of *E. faecium* used to spike product G at various levels. Download TABLE S4, DOCX file, 0.02 MB. Copyright © 2016 Patro et al. 2016 Patro et al. This content is distributed under the terms of the Creative Commons Attribution 4.0 International license.

10.1128/mSphere.00057-16.5 TABLE S5  Aggregate multicomponent overview including metagenomic sequencing run data obtained from the probiotics analyzed in this study. Download TABLE S5, DOCX file, 0.03 MB. Copyright © 2016 Patro et al. 2016 Patro et al. This content is distributed under the terms of the Creative Commons Attribution 4.0 International license.

10.1128/mSphere.00057-16.6 TABLE S6  Growth conditions for and morphologies of all of the species examined by medium type. Download TABLE S6, DOCX file, 0.02 MB. Copyright © 2016 Patro et al. 2016 Patro et al. This content is distributed under the terms of the Creative Commons Attribution 4.0 International license.

10.1128/mSphere.00057-16.7 TABLE S7  Medium-specific culture examination of the products in this study. Download TABLE S7, DOCX file, 0.02 MB. Copyright © 2016 Patro et al. 2016 Patro et al. This content is distributed under the terms of the Creative Commons Attribution 4.0 International license.
