# Supplementary material for: Culture-Independent Metagenomic Surveillance of Commercially Available Probiotics with High-Throughput Next-Generation Sequencing
Source: mSphere. 2016 Mar 30;1(2):e00057-16. doi: 10.1128/mSphere.00057-16 (PMC4894680; doi:10.1128/mSphere.00057-16)
Supplement: TABLE S1 [file sph002162055st1.docx]

**Table S1**

| **Accession** | **Bioproject** | **Sample** | **Library ID** |
| --- | --- | --- | --- |
| SRR2182530 | PRJNA291686 | SAMN03952426 | Product A- Lot 1 |
| SRR2182531 | PRJNA291686 | SAMN03952426 | Product A- Lot 2 |
| SRR2182542 | PRJNA291686 | SAMN03952426 | Product A- Lot 3 |
| SRR2182552 | PRJNA291686 | SAMN04009562 | Product B- Lot 1 |
| SRR2182553 | PRJNA291686 | SAMN04009562 | Product B- Lot 2 |
| SRR2182554 | PRJNA291686 | SAMN04009562 | Product B- Lot 3 |
| SRR2182555 | PRJNA291686 | SAMN04009563 | Product C- Lot 2 |
| SRR2182556 | PRJNA291686 | SAMN04009563 | Product C- Lot 3 |
| SRR2182557 | PRJNA291686 | SAMN04009564 | Product D- Lot 1 |
| SRR2182558 | PRJNA291686 | SAMN04009564 | Product D- Lot 2 |
| SRR2182532 | PRJNA291686 | SAMN04009564 | Product D- Lot 3 |
| SRR2182533 | PRJNA291686 | SAMN04009565 | Product E- Lot 1 |
| SRR2182534 | PRJNA291686 | SAMN04009565 | Product E- Lot 2 |
| SRR2182535 | PRJNA291686 | SAMN04009565 | Product E- Lot 3 |
| SRR2182536 | PRJNA291686 | SAMN04009566 | Product F- Lot 1 |
| SRR2182537 | PRJNA291686 | SAMN04009566 | Product F- Lot 2 |
| SRR2182538 | PRJNA291686 | SAMN04009566 | Product F- Lot 3 |
| SRR2182539 | PRJNA291686 | SAMN04009567 | Product G- Lot 1 |
| SRR2182540 | PRJNA291686 | SAMN04009567 | Product G- Lot 2 |
| SRR2182541 | PRJNA291686 | SAMN04009567 | Product G- Lot 3 |
| SRR2182543 | PRJNA291686 | SAMN04009568 | Product H- Lot 1 |
| SRR2182544 | PRJNA291686 | SAMN04009568 | Product H- Lot 2 |
| SRR2182545 | PRJNA291686 | SAMN04009568 | Product H- Lot 3 |
| SRR2182546 | PRJNA291686 | SAMN04009569 | Product I- Lot 1 |
| SRR2182547 | PRJNA291686 | SAMN04009569 | Product I- Lot 2 |
| SRR2182548 | PRJNA291686 | SAMN04009569 | Product I- Lot 3 |
| SRR2182549 | PRJNA291686 | SAMN04009570 | Product J- Lot 1 |
| SRR2182550 | PRJNA291686 | SAMN04009570 | Product J- Lot 2 |
| SRR2182551 | PRJNA291686 | SAMN04009570 | Product J- Lot 3 |
| SRR2191941 | PRJNA291686 | SAMN04012585 | Product G- Lot 1 *Enterococcus* Isolate |
| SRR2191942 | PRJNA291686 | SAMN04012586 | Product G- Lot 2 *Enterococcus* Isolate |
