# Supplementary material for: Culture-Independent Metagenomic Surveillance of Commercially Available Probiotics with High-Throughput Next-Generation Sequencing
Source: mSphere. 2016 Mar 30;1(2):e00057-16. doi: 10.1128/mSphere.00057-16 (PMC4894680; doi:10.1128/mSphere.00057-16)
Supplement: TABLE S2 [file sph002162055st2.docx]

**Table S2**

| **Genbank**  **Accession** | **Strain^a^** | **Target Name^b^** | **Signature Count^c^** | **Total Read Count^d^** | **Genome Size^e^** |
| --- | --- | --- | --- | --- | --- |
| NC_003909 | *Bacillus cereus* ATCC 10987 | *Bacillus cereus* | 37787 | 1976640 | 5223909 |
| NC_004722 | *Bacillus cereus* ATCC 14579 |  |  | 1975070 | 5411809 |
| CP002472 | *Bacillus coagulans* 2-6 | *Bacillus coagulans* | 47534 | 2064879 | 3073078 |
| CP003056 | *Bacillus coagulans* 36D1 |  |  | 2071868 | 3552226 |
| ABRX01 | *Bacillus pumilus* ATCC 7061 |  |  | 2500748 | 3833998 |
| NC_009848 | *Bacillus pumilus* SAFR-032 | *Bacillus pumilus* | 54745 | 2509741 | 3704465 |
| CP002906 | *Bacillus subtilis subsp. subtilis* RO-NN-1 | *Bacillus subtilis* | 50816 | 2646311 | 4011948 |
| ABQK01 | *Bacillus subtilis subsp. subtilis* str. 168 |  |  | 2645580 | 4214351 |
| CM000489 | *Bacillus subtilis subsp. subtilis str.* JH642 |  |  | 2624185 | 4187310 |
| ABQL01 | *Bacillus subtilis subsp. subtilis str.* NCIB 3610 |  |  | 2645368 | 4292752 |
| ABQN01 | *Bacillus subtilis subsp. subtilis str.* SMY |  |  | 2642916 | 4214338 |
| ABZX01 | *Bacteroides fragilis* 3_1_12 |  |  | 729906 | 5486239 |
| FQ312004 | *Bacteroides fragilis* 638R |  |  | 1002595 | 5373121 |
| AGXK01 | *Bacteroides fragilis* CL03T00C08 |  |  | 1012275 | 5225835 |
| AGXL01 | *Bacteroides fragilis* CL03T12C07 |  |  | 1004545 | 5214028 |
| AGXO01 | *Bacteroides fragilis* CL05T00C42 |  |  | 986344 | 5279416 |
| AGXP01 | *Bacteroides fragilis* CL05T12C13 |  |  | 976501 | 5272458 |
| AGXM01 | *Bacteroides fragilis* CL07T00C01 |  |  | 995516 | 5414224 |
| AGXN01 | *Bacteroides fragilis* CL07T12C05 |  |  | 1006093 | 5430227 |
| AGXQ01 | *Bacteroides fragilis* HMW 610 |  |  | 704671 | 5523030 |
| AGXR01 | *Bacteroides fragilis* HMW 615 |  |  | 992691 | 5358243 |
| ALOB01 | *Bacteroides fragilis* HMW 616 |  |  | 830236 | 5416584 |
| NC_003228 | *Bacteroides fragilis* NCTC 9343 | *Bacteroides fragilis* | 20845 | 1014445 | 5205140 |
| NC_006347 | *Bacteroides fragilis* YCH46 |  |  | 1002714 | 5277274 |
| NC_008618 | *Bifidobacterium adolescentis* ATCC 15703 | *Bifidobacterium adolescentis* | 47337 | 1537553 | 2089644 |
| AAXD02 | *Bifidobacterium adolescentis* L2-32 |  |  | 1535819 | 2385710 |
| CP002567 | *Bifidobacterium animalis subsp. animalis* ATCC 25527 |  |  | 1360017 | 1932693 |
| NC_011835 | *Bifidobacterium animalis subsp. lactis* AD011 | *Bifidobacterium animalis* | 74717 | 1925751 | 1933695 |
| NC_017866 | *Bifidobacterium animalis subsp. lactis* B420 |  |  | 1924854 | 1938595 |
| NC_017214 | *Bifidobacterium animalis subsp. lactis* BB-12 |  |  | 1927537 | 1942198 |
| NC_017867 | *Bifidobacterium animalis subsp. lactis* Bi-07 |  |  | 1925077 | 1938822 |
| NC_012814 | *Bifidobacterium animalis subsp. lactis* Bl-04 |  |  | 1924992 | 1938708 |
| NC_017216 | *Bifidobacterium animalis subsp. lactis* BLC1 |  |  | 1929876 | 1943990 |
| CP002915 | *Bifidobacterium animalis subsp. lactis* CNCM I-2494 |  |  | 1928988 | 1943113 |
| NC_012815 | *Bifidobacterium animalis subsp. lactis* DSM 10140 |  |  | 1924723 | 1938480 |
| ABOT01 | *Bifidobacterium animalis subsp. lactis* HN019 |  |  | 1903048 | 1915892 |
| CP001892 | *Bifidobacterium animalis subsp. lactis* V9 |  |  | 1930020 | 1944050 |
| CP001361 | *Bifidobacterium bifidum* BGN4 | *Bifidobacterium bifidum* | 66219 | 1991129 | 2223664 |
| AMPM01 | *Bifidobacterium bifidum* IPLA 20015 |  |  | 1916721 | 2138334 |
| AMPL01 | *Bifidobacterium bifidum* LMG 13195 |  |  | 1931783 | 2283145 |
| ABQP01 | *Bifidobacterium bifidum* NCIMB 41171 |  |  | 1957408 | 2186123 |
| CP001840 | *Bifidobacterium bifidum* PRL2010 |  |  | 1967280 | 2214656 |
| CP002220 | *Bifidobacterium bifidum* S17 |  |  | 1953034 | 2186882 |
| CP000246 | *Clostridium perfringens* ATCC 13124 |  |  | 2114343 | 3256683 |
| ABDV01 | *Clostridium perfringens* B str. ATCC 3626 |  |  | 2160109 | 3896305 |
| ABDU01 | *Clostridium perfringens* C str. JGS1495 |  |  | 2154549 | 3661329 |
| ABDX01 | *Clostridium perfringens* CPE str. F4969 |  |  | 2142931 | 3510272 |
| ABOO01 | *Clostridium perfringens* D str. JGS1721 |  |  | 2130094 | 4045016 |
| ABDW01 | *Clostridium perfringens* E str. JGS1987 |  |  | 2121363 | 4127102 |
| AFES01 | *Clostridium perfringens* F262 |  |  | 2076611 | 3464252 |
| ABDY01 | *Clostridium perfringens* NCTC 8239 |  |  | 2140347 | 3324319 |
| CP000312 | *Clostridium perfringens* SM101 | *Clostridium perfringens* | 56910 | 2251742 | 2897393 |
| NC_003366 | *Clostridium perfringens* str. 13 |  |  | 2080196 | 3031430 |
| ADLP01 | *Clostridium perfringens* WAL-14572 |  |  | 2095367 | 3462146 |
| AJGS01 | *Clostridium thermocellum* AD2 |  |  | 2840749 | 3433572 |
| NC_009012 | *Clostridium thermocellum* ATCC 27405 | *Clostridium thermocellum* | 65971 | 3058243 | 3843301 |
| CP002416 | *Clostridium thermocellum* DSM 1313 |  |  | 2961762 | 3561619 |
| ACVX01 | *Clostridium thermocellum* DSM 2360 |  |  | 2859027 | 3454605 |
| ABVG02 | *Clostridium thermocellum* JW20 |  |  | 3057112 | 3767533 |
| AJGT01 | *Clostridium thermocellum* YS ctg98 |  |  | 2867879 | 3464366 |
| NC_010816 | *Bifidobacterium long*um DJO10A | *Bifidobacterium longum* | 32599 | 1316884 | 2375792 |
| NC_004307 | *Bifidobacterium longum* NCC2705 |  |  | 1292406 | 2256640 |
| NC_015052 | *Bifidobacterium longum subsp. infantis* 157F |  |  | 1320398 | 2400312 |
| NC_011593 | *Bifidobacterium longum subsp. infantis* ATCC 15697 |  |  | 978924 | 2832748 |
| ABQQ01 | *Bifidobacterium longum subsp. infantis* CCUG 52486 |  |  | 1270438 | 2453368 |
| AP010889 | *Bifidobacterium longum subsp. infantis* JCM 1222 DNA |  |  | 979073 | 2828958 |
| AJTF01 | *Bifidobacterium longum subsp. longum* 1-6B |  |  | 1278637 | 2686768 |
| AJTJ01 | *Bifidobacterium longum subsp. longum* 2-2B |  |  | 1287179 | 2625691 |
| AJTI01 | *Bifidobacterium longum subsp. longum* 35B |  |  | 1284557 | 2514425 |
| AJTM01 | *Bifidobacterium longum subsp. longum* 44B |  |  | 1277290 | 2559215 |
| ACHI01 | *Bifidobacterium longum subsp. longum* ATCC 55813 |  |  | 1269246 | 2372858 |
| CP002286 | *Bifidobacterium longum subsp. longum* BBMN68 |  |  | 1277265 | 2265943 |
| FP929034 | *Bifidobacterium longum subsp. longum* F8 |  |  | 1263790 | 2372585 |
| AP010888 | *Bifidobacterium longum subsp. longum JCM* 1217 |  |  | 1293933 | 2385164 |
| CP002010 | *Bifidobacterium longum subsp. longum* JDM301 |  |  | 1171308 | 2477838 |
| CP002794 | *Bifidobacterium longum subsp. longum* KACC 91563 |  |  | 1292779 | 2385298 |
| CP000726 | *Clostridium botulinum A str.* ATCC 19397 |  |  | 722374 | 3863450 |
| AM412317 | *Clostridium botulinum A str*. ATCC 3502 | *Clostridium botulinum A* | 12022 | 726621 | 3886916 |
| CP000727 | *Clostridium botulinum A str.* Hall |  |  | 710940 | 3760560 |
| CP001581 | *Clostridium botulinum A2 str.* Kyoto |  |  | 627723 | 4155278 |
| CP000939 | *Clostridium botulinum B1 str.* Okra |  |  |  |  |
| AMXI01 | *Clostridium botulinum* CFSAN001627 |  |  | 656974 | 4076805 |
| AMXJ02 | *Clostridium botulinum* CFSAN001628 |  |  | 602012 | 4013630 |
| CP002011 | *Clostridium botulinum* F str. 230613 |  |  | 671411 | 3993072 |
| CP000728 | *Clostridium botulinum* F str. Langeland |  |  | 672782 | 3995377 |
| FR773526 | *Clostridium botulinum* H04402 065 |  |  | 643430 | 3919740 |
| ABDO02 | *Clostridium botulinum* NCTC 2916 |  |  | 638674 | 4031357 |
| ACAK01 | *Enterococcus faecalis* ARO1/DG |  |  | 2341226 | 2821088 |
| ACAG01 | *Enterococcus faecalis* ATCC 4200 |  |  | 2391328 | 3008845 |
| CP002621 | *Enterococcus faecalis* OG1RF | *Enterococcus faecalis* | 79578 | 2446128 | 2739625 |
| ACAD01 | *Enterococcus faecalis* T1 |  |  | 2412937 | 2905627 |
| ACAU01 | *Enterococcus faecalis* T11 |  |  | 2398243 | 2729086 |
| ACOC01 | *Enterococcus faecalis* T8 |  |  | 2391429 | 2985407 |
| ACOX02 | *Enterococcus faecalis* TUSoD |  |  | 2407787 | 2836620 |
| AE016830 | *Enterococcus faecalis* V583 |  |  | 2409564 | 3218031 |
| ACAW01 | *Enterococcus faecalis* X98 |  |  |  |  |
| ACAZ01 | *Enterococcus faecium* 1,141,733 |  |  | 1372158 | 2865114 |
| ACBA01 | *Enterococcus faecium* 1,231,410 |  |  | 2308521 | 2943808 |
| CP003351 | *Enterococcus faecium* Aus0004 | *Enterococcus faecium* | 84870 | 2432977 | 2955294 |
| ACBC01 | *Enterococcus faecium* Com12 |  |  | 1359603 | 2685402 |
| ACZZ01 | *Enterococcus faecium* D344SRF |  |  | 2200187 | 2745330 |
| CP003583 | *Enterococcus faecium* DO |  |  | 2371183 | 2698137 |
| CP001107 | *Eubacterium rectale* ATCC 33656 | *Eubacterium rectale* | 85728 | 2578756 | 3449685 |
| FP929042 | *Eubacterium rectale* DSM 17629 |  |  | 2397936 | 3259030 |
| FP929043 | *Eubacterium rectale* M104/1 |  |  | 2368796 | 3566572 |
| ADLZ01 | *Fusobacterium necrophorum subsp. funduliforme* 1_1_36S |  |  | 1799363 | 2300850 |
| AJSY01 | *Fusobacterium necrophorum subsp. funduliforme* ATCC 51357 | *Fusobacterium necrophorum* | 65732 | 1866876 | 2110802 |
| ALKK01 | *Fusobacterium necrophorum subsp. funduliforme Fnf* 1007 |  |  | 1705891 | 2166822 |
| ACHN01 | *Lactobacillus acidophilus* ATCC 4796 |  |  | 1908875 | 1999662 |
| CBLQ01 | *Lactobacillus acidophilus* CIP 76.13 |  |  | 1909485 | 1949435 |
| CBLP01 | *Lactobacillus acidophilus* CIRM-BIA 442 |  |  | 1939113 | 1986992 |
| CBLR01 | *Lactobacillus acidophilus* CIRM-BIA 445 |  |  | 1913436 | 1998365 |
| CBLT01 | *Lactobacillus acidophilus* DSM 20242 |  |  | 1953295 | 2047860 |
| CBLS01 | *Lactobacillus acidophilus* DSM 9126 |  |  | 1902892 | 1988965 |
| NC_021181 | *Lactobacillus acidophilus* La-14 |  |  | 1941286 | 1991579 |
| CP000033 | *Lactobacillus acidophilus* NCFM | *Lactobacillus acidophilus* | 71298 | 1941608 | 1993560 |
| NC_009089 | *Clostridium difficile* 630 | *Clostridium difficile* | 63459 | 3088547 | 4290252 |
| NC_013315 | *Clostridium difficile* CD196 |  |  | 3073266 | 4110554 |
| NC_013316 | *Clostridium difficile* R20291 |  |  | 3081230 | 4191139 |
| CP000416 | *Lactobacillus brevis* ATCC 367 | *Lactobacillus brevis* | 88585 | 2255504 | 2291220 |
| ACGH01 | *Lactobacillus buchneri* ATCC 11577 |  |  | 1184 | 2858739 |
| CP003043 | *Lactobacillus buchneri* CD034 |  |  | 1995765 | 2500564 |
| CP002652 | *Lactobacillus buchneri* NRRL B-30929 | *Lactobacillus buchneri* | 53653 | 1997226 | 2506301 |
| AFYJ01 | *Lactobacillus casei* 12A |  |  | 1941699 | 2885553 |
| AFYK01 | *Lactobacillus casei* 21/1 |  |  | 1947371 | 3215619 |
| AFYL01 | *Lactobacillus casei* 32G |  |  | 1922614 | 3011278 |
| AFYM01 | *Lactobacillus casei* A2-362 |  |  | 1936857 | 3360883 |
| CP000423 | *Lactobacillus casei* ATCC 334 | *Lactobacillus casei group* | 60168 | 1964209 | 2895264 |
| CP002618 | *Lactobacillus casei* BD-II |  | 34843 | 1956333 | 3069925 |
| FM177140 | *Lactobacillus casei* BL23 |  |  | 1956753 | 3079196 |
| AFYN01 | *Lactobacillus casei* CRF28 |  |  | 1936283 | 3036269 |
| AFYT01 | *Lactobacillus casei* Lc-10 |  |  | 1926939 | 2951277 |
| CP002616 | *Lactobacillus casei* LC2W |  |  | 1956496 | 3039042 |
| AQPP01 | *Lactobacillus casei* LcA |  |  | 1958694 | 3126576 |
| ARNV01 | *Lactobacillus casei LcY* |  |  | 1962857 | 3141543 |
| CP005486 | *Lactobacillus casei* LOCK919 |  |  | 1944645 | 3113601 |
| AFYU01 | *Lactobacillus casei* Lpc-37 |  |  | 1940417 | 3075227 |
| AFYO01 | *Lactobacillus casei* M36 |  |  | 1942548 | 3151936 |
| CP001084 | *Lactobacillus casei str.* Zhang |  |  | 1945605 | 2861848 |
| AFYP01 | *Lactobacillus casei* T71499 |  |  | 1931028 | 2999829 |
| AFYQ01 | *Lactobacillus casei* UCD174 |  |  | 1923810 | 3070430 |
| AFYR01 | *Lactobacillus casei* UW1 |  |  | 1902683 | 2865343 |
| AFYS01 | *Lactobacillus casei* UW4 |  |  | 1852708 | 2758163 |
| HE970764 | *Lactobacillus casei* W56 |  |  | 1956446 | 3075779 |
| CP002391 | *Lactobacillus paracasei subsp. paracasei* 8700:2 |  |  | 1948011 | 2939026 |
| ACGY01 | *Lactobacillus paracasei subsp. paracasei* ATCC 25302 |  |  | 1928533 | 2885585 |
| ANJX01 | *Lactobacillus paracasei subsp. paracasei* CNCM I-4270 |  |  | 1894820 | 2987345 |
| ANKH01 | *Lactobacillus paracasei subsp. paracasei* CNCM I-4649 |  |  | 1868186 | 2980999 |
| AP012541 | *Lactobacillus paracasei subsp. paracasei* JCM 8130 |  |  | 1942693 | 2995875 |
| ANMK01 | *Lactobacillus paracasei subsp. paracasei* Lpp120 |  |  | 1935717 | 2801291 |
| ANJZ01 | *Lactobacillus paracasei subsp. paracasei* Lpp14 |  |  | 1908643 | 2883109 |
| ANKA01 | *Lactobacillus paracasei subsp. paracasei* Lpp228 |  |  | 1932845 | 2936040 |
| ACPV01 | *Lactobacillus crispatus* 125-2-CHN |  |  | 1322163 | 2130372 |
| ADGR | *Lactobacillus crispatus* 214-1 |  |  | 1311781 | 2068805 |
| ADML01 | *Lactobacillus crispatus* CTV-05 |  |  | 1273302 | 2299477 |
| AGZF01 | *Lactobacillus crispatus* FB049-03 |  |  | 1364957 | 2342850 |
| AGZG01 | *Lactobacillus crispatus* FB077-07 |  |  | 1365012 | 2526985 |
| ACKR01 | *Lactobacillus crispatus* JV-V01 |  |  | 1331959 | 2066562 |
| ACOG01 | *Lactobacillus crispatus* MV-1A-US |  |  | 1338732 | 2165890 |
| ACQC01 | *Lactobacillus crispatus* MV-3A-US |  |  | 1336873 | 2285212 |
| ADDT01 | *Lactobacillus crispatus* SJ-3C-US |  |  | 1351343 | 2087871 |
| FN692037 | *Lactobacillus crispatus* ST1 | *Lactobacillus crispatus* | 42400 | 1365893 | 2042570 |
| CP000156 | *Lactobacillus delbrueckii subsp. bulgaricus* 2038 |  |  | 1641764 | 1872918 |
| CR954253 | *Lactobacillus delbrueckii subsp. bulgaricus* ATCC 11842 | *Lactobacillus delbrueckii subsp. bulgaricus* | 56627 | 1692434 | 1864998 |
| CP000412 | *Lactobacillus delbrueckii subsp. bulgaricus* ATCC BAA-365 |  |  | 1658735 | 1856951 |
| AGHW01 | *Lactobacillus delbrueckii subsp. bulgaricus* CNCM I-1519 |  |  | 1566934 | 1796538 |
| AGFO01 | *Lactobacillus delbrueckii subsp. bulgaricus* CNCM I-1632 |  |  | 1559839 | 1767897 |
| CP002341 | *Lactobacillus delbrueckii subsp. bulgaricus* ND02 |  |  | 1470743 | 2125753 |
| AEAT01 | *Lactobacillus delbrueckii subsp. bulgaricus* PB2003/044-T3-4 |  |  | 1427708 | 1977342 |
| ACQG01 | *Lactobacillus fermentum* 28-3-CHN |  |  | 1673424 | 1958290 |
| ACGI01 | *Lactobacillus fermentum* ATCC 14931 |  |  | 1640227 | 1796948 |
| CP002033 | *Lactobacillus fermentum* CECT 5716 | *Lactobacillus fermentum* | 72844 | 1990265 | 2096775 |
| AP008937 | *Lactobacillus fermentum* IFO 3956 DNA |  |  | 1983131 | 2098684 |
| ACOZ01 | *Lactobacillus gasseri* 202-4 |  |  | 1495293 | 1820250 |
| ADFT01 | *Lactobacillus gasseri* 224-1 |  |  | 1690958 | 2005522 |
| CP000413 | *Lactobacillus gasseri* ATCC 33323 | *Lactobacillus gasseri* | 60457 | 1707677 | 1894360 |
| AKFQ01 | *Lactobacillus gasseri* CECT 5714 GAS000159 |  |  | 1628645 | 1907790 |
| ACGO02 | *Lactobacillus gasseri* JV-V03 |  |  | 826838 | 2011295 |
| ABWH02 | *Lactobacillus gasseri* MV-22 |  |  | 1699102 | 1929035 |
| ADDU01 | *Lactobacillus gasseri* SJ-9E-US |  |  | 1670558 | 1782000 |
| ADDY01 | *Lactobacillus gasseri* SV-16A-US |  |  | 1687061 | 1985958 |
| CP000517 | *Lactobacillus helveticus* DPC 4571 | *Lactobacillus helveticus* | 50002 | 1606399 | 2080931 |
| ACLM01 | *Lactobacillus helveticus* DSM 20075 |  |  | 1358289 | 1808667 |
| CP002429 | *Lactobacillus helveticus* H10 |  |  | 1451078 | 2145899 |
| AEYL01 | *Lactobacillus helveticus* MTCC 5463 |  |  | 1457316 | 2048433 |
| CP003799 | *Lactobacillus helveticus* R0052 |  |  | 1362607 | 2129203 |
| ABWG02 | *Lactobacillus jensenii* 1153 |  |  | 344231 | 1737886 |
| ACQN01 | *Lactobacillus jensenii* 115-3-CHN |  |  | 352733 | 1615210 |
| ACOY01 | *Lactobacillus jensenii* 269-3 |  |  | 337910 | 1688268 |
| ACOF01 | *Lactobacillus jensenii* 27-2-CHN |  |  | 351636 | 1614212 |
| CM000953 | *Lactobacillus jensenii* JV-V16 | *Lactobacillus jensenii* | 6754 | 363487 | 1600834 |
| ACQD01 | *Lactobacillus jensenii* SJ-7A-US |  |  | 340106 | 1676718 |
| ACGR01 | *Lactobacillus johnsonii* ATCC 33200 |  |  | 1191442 | 1772891 |
| CP002464 | *Lactobacillus johnsonii* DPC 6026 | *Lactobacillus johnsonii* | 42011 | 1470977 | 1966342 |
| FN298497 | *Lactobacillus johnsonii* FI9785 |  |  | 1149980 | 1755993 |
| AE017198 | *Lactobacillus johnsonii* NCC 533 |  |  | 1332168 | 1992676 |
| AFQJ | *Lactobacillus johnsonii* pf0*1* |  |  | 1339669 | 1882796 |
| CP001617 | *Lactobacillus plantarum* JDM1 | *Lactobacillus plantarum* | 50527 | 2374868 | 3197756 |
| ACGZ02 | *Lactobacillus plantarum subsp. plantarum* ATCC 14917 |  |  | 2235768 | 3198761 |
| AGRI01 | *Lactobacillus plantarum subsp. plantarum* NC8 |  |  | 2233553 | 3207211 |
| CP002222 | *Lactobacillus plantarum subsp. plantarum* ST-III |  |  | 2266178 | 3254376 |
| AL935263 | *Lactobacillus plantarum* WCFS1 |  |  | 2221683 | 3308273 |
| AAPZ02 | *Lactobacillus reuteri* 100-23 |  |  | 1008120 | 2305553 |
| CACS | *Lactobacillus reuteri* ATCC 53608 |  |  | 982106 | 1968532 |
| ACHG01 | *Lactobacillus reuteri* CF48-3A |  |  | 997823 | 2032595 |
| CP000705 | *Lactobacillus reuteri* DSM 20016 | *Lactobacillus reuteri* | 32513 | 1146264 | 1999618 |
| AP007281 | *Lactobacillus reuteri* JCM 1112 DNA |  |  | 1153050 | 2039414 |
| AEAX01 | *Lactobacillus reuteri lpuph* |  |  | 986926 | 2116617 |
| AEAW | *Lactobacillus reuteri* mlc3 |  |  | 908519 | 2018629 |
| ACLB01 | *Lactobacillus reuteri* MM2-3 |  |  | 1099816 | 1943465 |
| ACGX02 | *Lactobacillus reuteri* MM4-1A |  |  | 1161808 | 2067914 |
| CP002844 | *Lactobacillus reuteri* SD2112 |  |  | 1112983 | 2264398 |
| AFZY | *Lactobacillus rhamnosus* ATCC 21052 |  |  | 2012574 | 2873519 |
| AP011548 | *Lactobacillus rhamnosus* ATCC 53103 | *Lactobacillus rhamnosus* | 54371 | 2164056 | 3005051 |
| CP003094 | *Lactobacillus rhamnosus* ATCC 8530 |  |  | 2130452 | 2960339 |
| AFYD | *Lactobacillus rhamnosus* CASL |  |  | 2083491 | 2849911 |
| FM179322 | *Lactobacillus rhamnosus GG* (ATCC 53103) |  |  | 2167489 | 3010111 |
| ABWJ01 | *Lactobacillus rhamnosus* HN001 |  |  | 2057834 | 2874088 |
| FM179323 | *Lactobacillus rhamnosus* Lc 705 |  |  | 2131810 | 2968598 |
| ACIZ01 | *Lactobacillus rhamnosus* LMS2-1 |  |  | 2114833 | 3109802 |
| AMQW01 | *Lactobacillus rhamnosus* LRHMDP2 |  |  |  |  |
| AMQX01 | *Lactobacillus rhamnosus* LRHMDP3 |  |  | 1951602 | 2911934 |
| AGKC | *Lactobacillus rhamnosus* R0011 |  |  | 2032759 | 2900620 |
| AEBA01 | *Lactobacillus salivarius* ACS-116-V-Col5a |  |  | 1243728 | 2044600 |
| ACGT01 | *Lactobacillus salivarius* ATCC 11741 |  |  | 1249843 | 1995868 |
| CP002034 | *Lactobacillus salivarius* CECT 5713 | *Lactobacillus salivarius* | 37452 | 1353277 | 1826378 |
| AFOI01 | *Lactobacillus salivarius* GJ-24 |  |  | 1258957 | 1995968 |
| AFMN | *Lactobacillus salivarius* NIAS840 84_1 |  |  | 1251944 | 2045276 |
| AICL01 | *Lactobacillus salivarius* SMXD51 |  |  | 1280148 | 1967681 |
| CP000233 | *Lactobacillus salivarius* UCC118 |  |  | 1311374 | 1827111 |
| CP003132 | *Lactococcus lactis subsp. cremoris* A76 |  |  | 1732129 | 2452616 |
| AGHX01 | *Lactococcus lactis subsp. cremoris* CNCM I-1631 |  |  | 17733 | 2511320 |
| NC_009004 | *Lactococcus lactis subsp. cremoris* MG1363 | *Lactococcus lactis subsp. cremoris* | 54558 | 1874629 | 2529478 |
| CP002094 | *Lactococcus lactis subsp. cremoris* NZ9000 |  |  | 1872784 | 2530294 |
| NC_008527 | *Lactococcus lactis subsp. cremoris* SK11 |  |  | 1802945 | 2438589 |
| CP003157 | *Lactococcus lactis subsp. cremoris* UC509.9 |  |  | 1695413 | 2250427 |
| NC_010471 | *Leuconostoc citreum* KM20 | *Leuconostoc citreum* | 64324 | 1687815 | 1796284 |
| CAGE01 | *Leuconostoc citreum* LBAE C10 |  |  | 1600584 | 1934423 |
| CAGF01 | *Leuconostoc citreum* LBAE C11 |  |  |  |  |
| CAGG01 | *Leuconostoc citreum* LBAE E16 |  |  | 1630851 | 1801604 |
| CP000422 | *Pediococcus pentosaceus* ATCC 25745 | *Pediococcus pentosaceus* | 51670 | 1540793 | 1832387 |
| CAHU01 | *Pediococcus pentosaceus* IE-3 |  |  | 1534228 | 1802376 |
| CP002122 | *Prevotella melaninogenica* ATCC 25845 | *Prevotella melaninogenica* | 47814 | 2001034 | 3168282 |
| ACWY01 | *Prevotella melaninogenica* D18 |  |  | 1982436 | 3213439 |
| CP002403 | *Ruminococcus albus* 7 | *Ruminococcus albus* | 2843 | 197757 | 3685408 |
| AZAA01 | *Ruminococcus albus* 8 |  |  | 161790 | 4373729 |
| CP000024 | *Streptococcus thermophilus* CNRZ1066 | *Streptococcus thermophilus* | 64726 | 1737242 | 1796226 |
| FR875178 | *Streptococcus thermophilus* JIM 8232 |  |  | 1602944 | 1929905 |
| CP000419 | *Streptococcus thermophilus* LMD-9 |  |  | 1644350 | 1856368 |
| CP000023 | *Streptococcus thermophilus* LMG 18311 |  |  | 1712640 | 1796846 |
| AE014075 | *Escherichia coli* CFT073 |  |  | 3494000 | 5231148 |
| BA000007 | *Escherichia coli O157:H7 str.* Sakai DNA | *Escherichia coli* | 89295 | 3571694 | 5498450 |
| CP001363 | *Salmonella enterica subsp. enterica serovar Typhimurium str.* 14028S | *Salmonella Typhimurium* | 92200 | 4171183 | 4870265 |
| FN424405 | *Salmonella enterica subsp. enterica serovar Typhimurium str.* D23580 |  |  | 4141210 | 4879400 |
| ACWH01 | *Bacteroides ovatus* 3_8_47FAA |  |  | 1476346 | 6545232 |
| AAXF02 | *Bacteroides ovatus* ATCC 8483 | *Bacteroides ovatus group* | 37220 | 1491405 | 6463169 |
| AGXT01 | *Bacteroides ovatus* CL02T12C04 |  |  | 1484802 | 7880760 |
| AGXU01 | *Bacteroides ovatus* CL03T12C18 |  |  | 1484133 | 6972153 |
| ADMP01 | *Bacteroides ovatus* SD CC 2a |  |  | 610 | 6050197 |
| ADMO01 | *Bacteroides ovatus* SD CMC 3f |  |  | 1465402 | 6775277 |
| NC_004663 | *Bacteroides thetaiotaomicron* VPI-5482 |  |  | 19501 | 6260361 |
| NC_009614 | *Bacteroides vulgatus* ATCC 8482 | *Bacteroides vulgatus* | 79606 | 3125384 | 5163189 |
| AGXZ01 | *Bacteroides vulgatus* CL09T03C04 |  |  | 2818501 | 4900419 |
| ADKO01 | *Bacteroides vulgatus* PC510 |  |  | 2892319 | 4781699 |
| BACQ | *Lactobacillus zeae* KCTC 3804 | *Lactobacillus zeae* | 113644 | 2962158 | 3110326 |
| NC_003030 | *Clostridium acetobutylicum* ATCC 824 |  |  | 3430708 | 3940880 |
| CP002660 | *Clostridium acetobutylicum* DSM 1731 |  |  | 3433861 | 3942462 |
| CP002118 | *Clostridium acetobutylicum* EA 2018 | *Clostridium acetobutylicum* | 76713 | 3433752 | 3940230 |
| CP000673 | *Clostridium kluyveri* DSM 555 |  |  | 3410999 | 3964618 |
| AP009049 | *Clostridium kluyveri* NBRC 12016 | *Clostridium kluyveri* | 75074 | 3365981 | 3896121 |
| CP003325 | *Bifidobacterium asteroides* PRL2011 | *Bifidobacterium asteroides* | 85360 | 2159745 | 2167304 |
| CP002764 | *Lactobacillus kefiranofaciens* ZW3 | *Lactobacillus kefiranofaciens* | 66218 | 1837936 | 2113023 |
| CM001538 | *Lactobacillus pentosus* KCA1 | *Lactobacillus pentosus* | 129105 | 3364781 | 3418123 |
| CR936503 | *Lactobacillus sakei strain* 23K | *Lactobacillus sakei* | 70360 | 1835822 | 1884661 |
| CP002461 | *Lactobacillus sanfranciscensis* TMW 1.1304 | *Lactobacillus sanfranciscensis* | 48974 | 1280377 | 1298316 |
| CP001602 | *Listeria monocytogenes* 08-5578 |  |  |  |  |
| AARJ | *Listeria monocytogenes* FSL J1-194 |  |  | 2013531 | 2989818 |
| CP002816 | *Listeria monocytogenes* M7 (4a) |  |  | 1449046 | 2976163 |
| AADQ | *Listeria monocytogenes serotype 1/2a str.* F6854 |  |  | 1447963 | 2950285 |
| AADR | *Listeria monocytogenes serotype 4b str.* H7858 |  |  | 1986297 | 2972254 |
| AE017262 | *Listeria monocytogenes str.* F2365 (4b) | *Listeria monocytogenes* | 47126 | 2044093 | 2905187 |
| AL591824 | *Listeria monocytogenes strain* EGD |  |  | 1441723 | 2944528 |
| CP000159 | *Salinibacter ruber* DSM 13855 | *Salinibacter ruber* | 107610 | 3213302 | 3551823 |
| FP565814 | *Salinibacter ruber* M8 |  |  | 3207746 | 3619447 |
| CP003274 | *Alistipes finegoldii* DSM 17242 | *Alistipes sp.* | 13184 | 519245 | 3734239 |
| ADLD01 | *Alistipes indistinctus* YIT 12060 |  |  | 10992 | 2850978 |
| ABFK02 | *Alistipes putredinis* DSM 17216 |  |  | 83767 | 2549878 |
| FP929032 | *Alistipes shahii* WAL 8301 |  |  | 437279 | 3649672 |
| AAYH02 | *Bacteroides uniformis* ATCC 8492 | *Bacteroides uniformis* | 96866 | 2957264 | 4717497 |
| AGXX01 | *Bacteroides uniformis* CL03T00C23 |  |  | 2925754 | 4914943 |
| AGXY01 | *Bacteroides uniformis* CL03T12C37 |  |  | 2929764 | 4890738 |
| AGXE01 | *Bacteroides xylanisolvens* CL03T12C04 |  |  | 563690 | 6056103 |
| ADKP01 | *Bacteroides xylanisolvens* SD CC 1b |  |  | 8243 | 6059806 |
| FP929033 | *Bacteroides xylanisolvens* XB1A | *Bacteroides xylanisolvens* | 11167 | 566352 | 5800812 |
| ACDI02 | *Bacteroides dorei* 5_1_36/D4 |  |  | 3199772 | 5534748 |
| AGXH01 | *Bacteroides dorei* CL02T00C15 |  |  | 3317903 | 6010738 |
| AGXJ01 | *Bacteroides dorei* CL02T12C06 |  |  | 3311869 | 5997311 |
| AGXI01 | *Bacteroides dorei* CL03T12C01 |  |  | 3265730 | 5387249 |
| ABWZ01 | *Bacteroides dorei* DSM 17855 | *Bacteroides dorei* | 89082 | 3388520 | 5487733 |
| CP001056 | *Clostridium botulinum B str.* Eklund 17B | *Clostridium botulinum B* | 60030 | 2850165 | 3800327 |
| ALYJ01 | *Clostridium botulinum* CDC66177 |  |  | 2746069 | 3852437 |
| ACSC01 | *Clostridium botulinum E1 str.* 'BoNT E Beluga' |  |  | 1227658 | 3999201 |
| CP001078 | *Clostridium botulinum E3 str.* Alaska E43 |  |  | 1200713 | 3659644 |
| CP002410 | *Clostridium botulinum* BKT015925 | *Clostridium botulinum C* | 18496 | 1053840 | 2773157 |
| AESB01 | *Clostridium botulinum* BKT028387 |  |  | 956123 | 2833796 |
| ABDQ01 | *Clostridium botulinum C str.* Eklund |  |  | 290406 | 2961186 |
| AESA01 | *Clostridium botulinum C str.* Stockholm |  |  | 828958 | 2656036 |
| ACSJ01 | *Clostridium botulinum D str. 1873 CLG* |  |  | 887794 | 2379404 |
| AESC01 | *Clostridium botulinum V891* |  |  | 953264 | 3138929 |
| ALVD01 | *Fusobacterium nucleatum ChDC F128* |  |  | 223406 | 2361679 |
| AFQD01 | *Fusobacterium nucleatum subsp. animalis ATCC 51191* |  |  | 233689 | 2270170 |
| AGEH01 | *Fusobacterium nucleatum subsp. animalis F0419 strain OT 420* |  |  | 248092 | 2408755 |
| AKXI01 | *Fusobacterium nucleatum subsp. fusiforme ATCC 51190* |  |  | 257627 | 1837112 |
| ADVK01 | *Fusobacterium nucleatum subsp. nucleatum ATCC 23726* |  |  | 286505 | 2237033 |
| AE009951 | *Fusobacterium nucleatum subsp. nucleatum ATCC 25586* | *Fusobacterium nucleatum* | 4457 | 305445 | 2174499 |
| AARG01 | *Fusobacterium nucleatum subsp. polymorphum ATCC 10953* |  |  | 264189 | 2440231 |
| ADDB01 | *Fusobacterium nucleatum subsp. polymorphum F0401* |  |  | 263444 | 2459803 |
| AABF01 | *Fusobacterium nucleatum subsp. vincentii ATCC 49256* |  |  | 256950 | 2118074 |
| ADHG | *Lactobacillus iners AB-1* |  |  | 1049921 | 1287456 |
| AEPX | *Lactobacillus iners ATCC 55195* | *Lactobacillus iners* | 28902 | 1044277 | 1219250 |
| ACLN01 | *Lactobacillus iners DSM 13335* |  |  | 1037601 | 1269484 |
| AEHQ01 | *Lactobacillus iners LactinV 01V1-a* |  |  | 1026557 | 1294172 |
| AEHP01 | *Lactobacillus iners LactinV 03V1-b* |  |  | 999419 | 1303955 |
| AEHO01 | *Lactobacillus iners LactinV 09V1-c* |  |  | 1027265 | 1312334 |
| AEHN01 | *Lactobacillus iners LactinV 11V1-d* |  |  | 1025949 | 1310250 |
| AEKI01 | *Lactobacillus iners LEAF 2052A-d* |  |  | 1033569 | 1322320 |
| AEKH01 | *Lactobacillus iners LEAF 2053A-b* |  |  | 1033621 | 1368716 |
| AEKK01 | *Lactobacillus iners LEAF 3008A-a* |  |  | 1030194 | 1273041 |
| AEXP01 | *Lactobacillus iners SPIN 1401G* |  |  |  |  |
| AEHR01 | *Lactobacillus iners SPIN 2503V10-D* |  |  | 1031861 | 1283897 |
| AEXJ01 | *Lactobacillus iners UPII 143-D* |  |  | 1028239 | 1257577 |
| AEXK01 | *Lactobacillus iners UPII 60-B* |  |  | 1029936 | 1323613 |
| ACXB01 | *Pediococcus acidilactici 7_4* |  |  | 1017324 | 2010555 |
| AEEG01 | *Pediococcus acidilactici DSM 20284* | *Pediococcus acidilactici* | 26488 | 1173449 | 1929953 |
| AGKB01 | *Pediococcus acidilactici MA18/5M* |  |  | 1086105 | 1989583 |
| AFPX01 | *Prevotella nigrescens ATCC 33563* | *Prevotella nigrescens* | 62063 | 2037387 | 2612230 |
| ADGJ01 | *Prevotella nigrescens F0103* |  |  | 2075770 | 3021733 |
| AJVZ01 | *Prevotella bivia DSM 20514* | *Prevotella bivia* | 77391 | 2034289 | 2520138 |
| ADFO01 | *Prevotella bivia JCVIHMP010* |  |  | 1987323 | 2424431 |
| AEPD01 | *Prevotella buccae ATCC 33574* | *Prevotella buccae* | 87550 | 2727010 | 3222824 |
| ACRB01 | *Prevotella buccae D17* |  |  | 2724637 | 3297977 |
| AEXO01 | *Prevotella denticola CRIS 18C-A* |  |  | 2463720 | 3177783 |
| CP002589 | *Prevotella denticola F0289* | *Prevotella denticola* | 76198 | 2484740 | 2937589 |
| AFNE01 | *Ruminococcus flavefaciens 17* |  |  | 81588 | 3454940 |
| ACOK01 | *Ruminococcus flavefaciens FD-1* | *Ruminococcus flavefaciens* | 1600 | 93418 | 4573343 |
| CP002365 | *Lactococcus lactis subsp. lactis CV56* |  |  | 38746 | 2399458 |
| AE005176 | *Lactococcus lactis subsp. lactis Il1403* |  |  | 128672 | 2365589 |
| AP012281 | *Lactococcus lactis subsp. lactis IO-1* |  |  | 380738 | 2421471 |
| NC_013656 | *Lactococcus lactis subsp. lactis KF147* | *Lactococcus lactis subsp. lactis* | 8127 | 451921 | 2598144 |
| AFCC01 | *Lactococcus garvieae 21881* |  |  | 719903 | 2164291 |
| AFCD01 | *Lactococcus garvieae 8831* |  |  | 709489 | 2085919 |
| AP009332 | *Lactococcus garvieae ATCC 49156* | *Lactococcus garvieae* | 14462 | 762938 | 1950135 |
| AMQS01 | *Lactococcus garvieae DCC43* |  |  | 119288 | 2244387 |
| AMFD01 | *Lactococcus garvieae I113* |  |  | 435345 | 2178733 |
| AKFO01 | *Lactococcus garvieae IPLA 31405* |  |  | 738634 | 2052312 |
| AFHF01 | *Lactococcus garvieae UNIUD074* |  |  | 772104 | 2171405 |
| ADJN01 | *Peptostreptococcus anaerobius 653-L* | *Peptostreptococcus anaerobius* | 58346 | 1692906 | 2083087 |
| AMEL01 | *Peptostreptococcus anaerobius VPI 4330* |  |  | 1690200 | 1988071 |
| ACGS02 | *Lactobacillus ruminis ATCC 25644* |  |  | 1618614 | 2103640 |
| CP003032 | *Lactobacillus ruminis ATCC 27782* | *Lactobacillus ruminis* | 49424 | 1770236 | 2066647 |
| AFOJ | *Lactobacillus ruminis SPM0211 83_1* |  |  | 1648323 | 2172130 |
| ABIX02 | *Bifidobacterium dentium ATCC 27678* |  |  | 2602174 | 2642081 |
| AEEQ | *Bifidobacterium dentium ATCC 27679* |  |  | 2278973 | 2633776 |
| NC_013714 | *Bifidobacterium dentium Bd1* | *Bifidobacterium dentium* | 97911 | 2601231 | 2636365 |
| AEHJ01 | *Bifidobacterium dentium JCVIHMP022* |  |  | 2277371 | 2636465 |
| CP002743 | *Bifidobacterium breve ACS-071-V-Sch8b* |  |  | 1671068 | 2327492 |
| AFVV01 | *Bifidobacterium breve CECT 7263* |  |  | 1640544 | 2314390 |
| AFXX01 | *Bifidobacterium breve DPC 6330* |  |  | 1652272 | 2386221 |
| ACCG02 | *Bifidobacterium breve DSM 20213* |  |  | 1611671 | 2329986 |
| CP000303 | *Bifidobacterium breve UCC2003* | *Bifidobacterium breve* | 56216 | 1787121 | 2422684 |
| CP002559 | *Lactobacillus acidophilus 30SC* |  |  | 1530626 | 2078001 |
| CP002338 | *Lactobacillus amylovorus GRL 1112* | *Lactobacillus amylovorus group* | 53680 | 1606521 | 2067692 |
| CP002609 | *Lactobacillus amylovorus GRL1118* |  |  | 1522377 | 1894401 |
| NC_010655 | *Akkermansia muciniphila ATCC BAA-835* | *Akkermansia muciniphila* | 105800 | 2662429 | 2664102 |
| ABYO01 | *Anaerococcus lactolyticus ATCC 51172* |  |  | 33912 | 2177741 |
| AEXM01 | *Anaerococcus prevotii ACS-065-V-Col13* |  |  | 81900 | 1697099 |
| CP001708 | *Anaerococcus prevotii DSM 20548* | *Anaerococcus prevotii* | 2344 | 150635 | 1883067 |
| ACGC01 | *Anaerococcus tetradius ATCC 35098* |  |  | 81017 | 2103623 |
| ADCP01 | *Bilophila wadsworthia 3_1_6* | *Bilophila wadsworthia* | 82546 | 3189523 | 4374195 |
| CP001810 | *Butyrivibrio proteoclasticus B316* | *Butyrivibrio proteoclasticus* | 70204 | 3110460 | 3554795 |
| FP929038 | *Coprococcus catus GD/7* | *Coprococcus catus* | 62377 | 2791851 | 3463414 |
| CP003220 | *Desulfovibrio desulfuricans ND132* | *Desulfovibrio desulfuricans* | 20 | 3930 | 3858580 |
| CP001358 | *Desulfovibrio desulfuricans subsp. desulfuricans str. ATCC 27774* |  |  | 2163 | 2873437 |
| AP010904 | *Desulfovibrio magneticus RS-1* | *Desulfovibrio magneticus* | 35425 | 1973899 | 5248049 |
| ALAO01 | *Desulfovibrio magneticus str. Maddingley MBC34* |  |  | 1948424 | 4391518 |
| CP000527 | *Desulfovibrio vulgaris DP4* |  |  | 2555759 | 3462887 |
| CP002297 | *Desulfovibrio vulgaris RCH1* | *Desulfovibrio vulgaris* | 69658 | 3084907 | 3532052 |
| CP001197 | *Desulfovibrio vulgaris str. Miyazaki F* |  |  | 37940 | 4040304 |
| AE017285 | *Desulfovibrio vulgaris subsp. vulgaris str. Hildenborough* |  |  | 3100591 | 3570858 |
| ADLU01 | *Dorea formicigenerans 4_6_53AFAA* |  |  | 2128853 | 3825692 |
| AAXA02 | *Dorea formicigenerans ATCC 27755* | *Dorea formicigenerans* | 62948 | 2135740 | 3186031 |
| AECU01 | *Faecalibacterium cf. prausnitzii KLE1255* |  |  | 272806 | 2926561 |
| ACOP02 | *Faecalibacterium prausnitzii A2-165* |  |  | 215089 | 3080849 |
| FP929045 | *Faecalibacterium prausnitzii L2/6* | *Faecalibacterium prausnitzii* | 7843 | 419474 | 3248271 |
| ABED02 | *Faecalibacterium prausnitzii M21/2* |  |  | 326004 | 3126983 |
| FP929046 | *Faecalibacterium prausnitzii SL3/3* |  |  | 305949 | 3165875 |
| CP002571 | *Helicobacter pylori 2017* |  |  | 741344 | 1548238 |
| AP011940 | *Helicobacter pylori F16* |  |  | 992413 | 1575399 |
| CP003905 | *Helicobacter pylori Rif1* |  |  | 940337 | 1667841 |
| CP003473 | *Helicobacter pylori Shi169* | *Helicobacter pylori* | 23023 | 1095537 | 1616909 |
| CP002073 | *Helicobacter pylori SJM180* |  |  | 899941 | 1658050 |
| CP001582 | *Helicobacter pylori v225d* |  |  | 1031696 | 1588278 |
| ADMG01 | *Sutterella wadsworthensis 2_1_59BFAA* |  |  | 51556 | 2665453 |
| ADMF01 | *Sutterella wadsworthensis 3_1_45B* | *Sutterella wadsworthensi* | 967 | 49974 | 2958116 |
| AAXG02 | *Pseudoflavonifractor capillosus ATCC 29799* | *Pseudoflavonifractor capillosus* | 81725 | 3644166 | 4241853 |
| ABYJ02 | *Roseburia intestinalis L1-82* |  |  | 3108893 | 4380675 |
| FP929049 | *Roseburia intestinalis M50/1* | *Roseburia intestinali* | 116226 | 3254391 | 4060667 |
| FP929050 | *Roseburia intestinalis XB6B4* |  |  | 3059761 | 4164855 |
| FP929054 | *Ruminococcus (Blautia) obeum A2-162* | *Blautia obeum* | 8574 | 398723 | 3692226 |
| AAVO02 | *Ruminococcus (Blautia) obeum ATCC 29174* |  |  | 400858 | 3624708 |
| AAVP02 | *Ruminococcus (Blautia) torques ATCC 27756* |  |  | 11313 | 2739406 |
| FP929055 | *Ruminococcus (Blautia) torques L2-14* | *Blautia torques* | 109 | 25645 | 3277778 |
| AECM01 | *Finegoldia magna ACS-171-V-Col3* |  |  | 1324826 | 1828755 |
| AP008971 | *Finegoldia magna ATCC 29328* | *Finegoldia magna* | 41549 | 1433855 | 1797577 |
| ACHM02 | *Finegoldia magna ATCC 53516* |  |  | 577592 | 1908011 |
| AEDP01 | *Finegoldia magna BVS033A4* |  |  | 1327258 | 1995204 |
| AFUI01 | *Finegoldia magna SY403409CC001050417* |  |  | 1353333 | 2032717 |
| AMBZ01 | *Paenibacillus alvei DSM 29* |  |  | 967898 | 6834602 |
| CFSAN001086 | *Paenibacillus alvei TS-15* | *Paenibacillus alvei* | 16235 | 939364 | 6713419 |
| AFOX01 | *Paenibacillus polymyxa ATCC 842* |  |  | 1159932 | 5898380 |
| CP000154 | *Paenibacillus polymyxa E681* | *Paenibacillus polymyxa* | 25161 | 1604576 | 5394884 |
| HE577054 | *Paenibacillus polymyxa M1* |  |  | 1261191 | 5864546 |
| CP003235 | *Paenibacillus mucilaginosus 3016* | *Paenibacillus mucilaginosus* | 62361 | 3991944 | 8739048 |
| CP003422 | *Paenibacillus mucilaginosus K02* |  |  | 3528922 | 8770140 |
| CP002869 | *Paenibacillus mucilaginosus KNP414* |  |  | 3612991 | 8663821 |
| none | *Paenibacillus* | *Paenibacillus* | 1128 |  |  |
| AJLV01 | *Bacillus licheniformis 10-1-A* |  |  | 3993745 | 4317010 |
| AJLW01 | *Bacillus licheniformis 5-2-D* |  |  | 4010766 | 4161258 |
| CP005965 | *Bacillus licheniformis 9945A* |  |  | 2514912 | 4376305 |
| AVEZ01 | *Bacillus licheniformis CG-B52* |  |  | 4022923 | 4396534 |
| AMWQ01 | *Bacillus licheniformis CGMCC 3963* |  |  |  |  |
| AE017333 | *Bacillus licheniformis DSM 13 = ATCC 14580* | *Bacillus licheniformis* | 155819 | 4064834 | 4222645 |
| JMEA01 | *Leuconostoc lactis* |  |  |  |  |
| AEOR01 | *Leuconostoc lactis KCTC 3528 = DSM 20202* |  | 18195 |  |  |
| ACKV01 | *Leuconostoc mesenteroides subsp. cremoris ATCC 19254* |  | 11192 |  | 1638511 |
| JAUJ01 | *Leuconostoc mesenteroides subsp. cremoris T26* |  |  |  |  |
| ATAZ01 | *Leuconostoc mesenteroides subsp. cremoris TIFN8* |  |  |  |  |
| NC_008531 | *Leuconostoc mesenteroides subsp. mesenteroides ATCC 8293* | *Leuconostoc mesenteroides* | 18639 |  |  |
| CP003101 | *Leuconostoc mesenteroides subsp. mesenteroides J18* |  | 63038 | 1743112 | 1896561 |
| Strains Assessed for Signature Exclusivity and Specificity^f^ | | | | | |
| AEXN01 | *Anaerococcus hydrogenalis ACS-025-V-Sch4* | | | | |
| ABXA01 | *Anaerococcus hydrogenalis DSM 7454* | | | | |
| CAGV01 | *Anaerococcus obesiensis ph10* | | | | |
| ACXU01 | *Anaerococcus vaginalis ATCC 51170* | | | | |
| NC_006582 | *Bacillus clausii KSM-K16* | | | | |
| AAVM02 | *Bacteroides caccae ATCC 43185* | | | | |
| AGXF01 | *Bacteroides caccae CL03T12C61* | | | | |
| AGXG01 | *Bacteroides cellulosilyticus CL02T12C19* | | | | |
| ACCH01 | *Bacteroides cellulosilyticus DSM 14838* | | | | |
| AFBM01 | *Bacteroides clarus YIT 12056* | | | | |
| ABIY02 | *Bacteroides coprocola DSM 17136* | | | | |
| ACBW01 | *Bacteroides coprophilus DSM 18228* | | | | |
| CM001167 | *Bacteroides coprosuis DSM 18011* | | | | |
| ACWG01 | *Bacteroides eggerthii 1_2_48FAA* | | | | |
| ABVO01 | *Bacteroides eggerthii DSM 20697* | | | | |
| AGDG01 | *Bacteroides faecis MAJ27* | | | | |
| AGXW01 | *Bacteroides finegoldii CL09T03C10* | | | | |
| ABXI02 | *Bacteroides finegoldii DSM 17565* | | | | |
| AFBN01 | *Bacteroides fluxus YIT 12057* | | | | |
| CP002352 | *Bacteroides helcogenes P 36-108* | | | | |
| ABJL02 | *Bacteroides intestinalis DSM 17393* | | | | |
| AGXS01 | *Bacteroides nordii CL02T12C05* | | | | |
| ABVQ01 | *Bacteroides pectinophilus ATCC 43243* | | | | |
| ABQC02 | *Bacteroides plebeius DSM 17135* | | | | |
| CP002530 | *Bacteroides salanitronis DSM 18170* | | | | |
| AGXV01 | *Bacteroides salyersiae CL02T12C01* | | | | |
| ABFZ02 | *Bacteroides stercoris ATCC 43183* | | | | |
| ABYS02 | *Bifidobacterium angulatum DSM 20098* | | | | |
| ABXY01 | *Bifidobacterium catenulatum DSM 16992* | | | | |
| ABXB03 | *Bifidobacterium gallicum DSM 20093* | | | | |
| ABXB03 | *Bifidobacterium gallicum DSM 20093* | | | | |
| ABXX02 | *Bifidobacterium pseudocatenulatum DSM 20438* | | | | |
| ADCO01 | *Bilophila sp. 4_1_30* | | | | |
| ABYU02 | *Blautia hansenii DSM 20583* | | | | |
| ACBZ01 | *Blautia hydrogenotrophica DSM 10507* | | | | |
| ABWN01 | *Butyrivibrio crossotus DSM 2876* | | | | |
| FP929036 | *Butyrivibrio fibrisolvens 16/4* | | | | |
| CP003326 | *Clostridium acidurici 9a* | | | | |
| BAEV01 | *Clostridium arbusti SL206* | | | | |
| ACCJ01 | *Clostridium asparagiforme DSM 15981* | | | | |
| ABEZ02 | *Clostridium bartlettii DSM 16795* | | | | |
| AKWA01 | *Clostridium beijerinckii G117* | | | | |
| ABCC02 | *Clostridium bolteae ATCC BAA-613* | | | | |
| CP000962 | *Clostridium botulinum A3 str. Loch Maree* | | | | |
| CP001083 | *Clostridium botulinum Ba4 str. 657* | | | | |
| ABDP01 | *Clostridium botulinum Bf* | | | | |
| ABDT01 | *Clostridium butyricum 5521* | | | | |
| ACOM01 | *Clostridium butyricum E4 str. BoNT E BL5262* | | | | |
| ADEK01 | *Clostridium carboxidivorans P7* | | | | |
| AMEZ01 | *Clostridium celatum DSM 1785* | | | | |
| CP001348 | *Clostridium cellulolyticum H10* | | | | |
| NC_014393 | *Clostridium cellulovorans 743B* | | | | |
| ADLJ01 | *Clostridium citroniae WAL-17108* | | | | |
| ACIO01 | *Clostridium hathewayi DSM 13479* | | | | |
| ABWP01 | *Clostridium hiranonis DSM 13275* | | | | |
| ABYI02 | *Clostridium hylemonae DSM 15053* | | | | |
| ABCB02 | *Clostridium leptum DSM 753* | | | | |
| ACEC01 | *Clostridium methylpentosum DSM 5476* | | | | |
| ABWO01 | *Clostridium nexile DSM 1787* | | | | |
| CP002109 | *Clostridium saccharolyticum WM1* | | | | |
| FP929037 | *Clostridium saccharolyticum-like K10* | | | | |
| ABFY02 | *Clostridium scindens ATCC 35704* | | | | |
| ABKW02 | *Clostridium sporogenes ATCC 15579* | | | | |
| AGAH01 | *Clostridium sporogenes PA 3679* | | | | |
| ABVR01 | *Coprococcus comes ATCC 27758* | | | | |
| ABEY02 | *Coprococcus eutactus ATCC 27759* | | | | |
| CP002431 | *Desulfovibrio aespoeensis Aspo-2* | | | | |
| CP003221 | *Desulfovibrio africanus str. Walvis Bay* | | | | |
| CP000112 | *Desulfovibrio alaskensis G20* | | | | |
| AECZ01 | *Desulfovibrio fructosovorans JJ* | | | | |
| FO203522 | *Desulfovibrio hydrothermalis AM13 DSM 14728* | | | | |
| ABXU01 | *Desulfovibrio piger ATCC 29098* | | | | |
| CP001649 | *Desulfovibrio salexigens DSM 2638* | | | | |
| AAXB02 | *Dorea longicatena DSM 13814* | | | | |
| CM001487 | *Eubacterium cellulosolvens 6* | | | | |
| FP929041 | *Eubacterium cylindroides* T2-87 | | | | |
| CP001104 | *Eubacterium eligens* ATCC 27750 | | | | |
| ACEP01 | *Eubacterium hallii* DSM 3353 | | | | |
| AGWI01 | *Eubacterium infirmum* F0142 | | | | |
| CP002273 | *Eubacterium limosum* KIST612 | | | | |
| AAVL02 | *Eubacterium ventriosum* ATCC 27560 | | | | |
| ACET01 | *Fusobacterium gonidiaformans* ATCC 25563 | | | | |
| ACDB02 | *Fusobacterium mortiferum* ATCC 9817 | | | | |
| ACJY01 | *Fusobacterium periodonticum* ATCC 33693 | | | | |
| ACIF01 | *Fusobacterium periodonticum* D10 | | | | |
| ACDH02 | *Fusobacterium ulcerans* ATCC 49185 | | | | |
| ACIE02 | *Fusobacterium varium* ATCC 27725 | | | | |
| CAGP01 | *Helicobacter bizzozeronii* CCUG 35545 | | | | |
| CP003479 | *Helicobacter cetorum* MIT 00-7128 | | | | |
| ABQU01 | *Helicobacter pullorum* MIT 98-5489 | | | | |
| BACS01 | *Lactobacillus acidipiscis* KCTC 13900 | | | | |
| ADNY01 | *Lactobacillus amylolyticus* DSM 11664 | | | | |
| AEOF01 | *Lactobacillus animalis* KCTC 3501 | | | | |
| ACLL01 | *Lactobacillus antri DSM 16041* | | | | |
| ACGG01 | *Lactobacillus brevis subsp. gravesensis* ATCC 27305 | | | | |
| AGEJ01 | *Lactobacillus catenaformis* OT 569 | | | | |
| ACOH01 | *Lactobacillus coleohominis* 101-4-CHN | | | | |
| AGBU01 | *Lactobacillus curvatus* CRL 705 | | | | |
| AICN01 | *Lactobacillus gastricus* PS3 | | | | |
| ACGP01 | *Lactobacillus hilgardii* ATCC 8290 | | | | |
| BACN01 | *Lactobacillus malefermentans* KCTC 3548 | | | | |
| AFTL01 | *Lactobacillus oris* F0423 | | | | |
| AEKL01 | *Lactobacillus oris* PB013-T2-3 | | | | |
| ACGU01 | *Lactobacillus ultunensis* DSM 16047 | | | | |
| ACGV01 | *Lactobacillus vaginalis* ATCC 49540 | | | | |
| FN822744 | *Leuconostoc gasicomitatum* LMG 18811 | | | | |
| ACCR | *Listeria grayi* DSM 20601 | | | | |
| AGCN | *Listeria innocua* ATCC 33091 | | | | |
| FR687253 | *Listeria ivanovii subsp. ivanovii* PAM 55 | | | | |
| AARF01 | *Paenibacillus larvae subsp. larvae* BRL-230010 | | | | |
| CP003107 | *Paenibacillus terrae* HPL-003 | | | | |
| CP003137 | *Pediococcus claussenii* ATCC BAA-344 | | | | |
| BANK01 | *Pediococcus lolii* NGRI 0510Q | | | | |
| ADGQ | *Peptostreptococcus stomatis* DSM 17678 | | | | |
| AFXP01 | *Prevotella histicola* F0411 | | | | |
| CP003502 | *Prevotella intermedia* 17 | | | | |
| AGEK01 | *Prevotella maculosa* OT 289 | | | | |
| AGWK01 | *Prevotella micans* F0438 | | | | |
| ADGI01 | *Prevotella oulorum* F0390 | | | | |
| CP003040 | *Roseburia hominis* A2-183 | | | | |
| ACFY01 | *Roseburia inulinivorans* DSM 16841 | | | | |
| AAYG02 | *Ruminococcus (Blautia) gnavus* ATCC 29149 | | | | |
| FP929051 | *Ruminococcus bromii* L2-63 | | | | |
| ABOU02 | *Ruminococcus lactaris* ATCC 29176 | | | | |
| AECT01 | *Streptococcus anginosus* F0211 | | | | |
| AFUP01 | *Streptococcus constellatus subsp. pharyngis* SK1060 | | | | |
| NC_018089 | *Streptococcus mutans* GS-5 | | | | |
| AFBQ01 | *Sutterella parvirubra* YIT 11816 | | | | |

1. Sequenced strains included in database for extraction of unique signatures under target genus species name in b.
2. Name specified in k-mer analysis output.
3. Number of k-mer signatures unique to specified bacterial name in b.
4. Number of possible 150 bp reads from respective genome that match species-specific k-mers in c.
5. In bp.
6. Genus and species genomes closely related to database construction important for assessing taxonomic specificity. Note list is not exhaustive for exclusivity testing of other diverse bacteria.
