# Supplementary material for: Culture-Independent Metagenomic Surveillance of Commercially Available Probiotics with High-Throughput Next-Generation Sequencing
Source: mSphere. 2016 Mar 30;1(2):e00057-16. doi: 10.1128/mSphere.00057-16 (PMC4894680; doi:10.1128/mSphere.00057-16)
Supplement: TABLE S3 [file sph002162055st3.docx]

**Table S3**

| **Organism** | **Gene** | **Forward Sequence^a^** | **Reverse Sequence^a^** |
| --- | --- | --- | --- |
| *B. animalis subsp. animalis* | *clpC* | GCGTGCACGTTACGAGAATC | AAACAGCCTCATCCTGACCG |
| *B. animalis subsp. lactis* | *clpC* | ACCGCAACCTTCCCGATAAG | TTGAGTCCCACACGTGTACG |
| *B. breve* | *clpC* | CGAGAACCACCATCACGTGA | CTTCTTGGACTCAGCCTGGG |
| *B. bifidum* | *fusA* | TGCTTCTGGAACCGTCAGTC | CCGAGCTTGTCCTTGATGGT |
| *B. longum subsp. longum* | *clpC* | TCAGGCCGAGTCCAAGAAAC | CTCGGACATATCCACGCGAA |
| *B. longum subsp. infantis* | *fusA* | GGCATCACCATCCAGTCCG | CCTGCATCTCGACCAGGTC |
| *L. acidophilus* | *leuS* | ACGGTACAGGTGCAGTGATG | TGAAGTCCCAGTCACGAAGC |
| *L. casei / paracasei* | *fusA* | ACCATCACCAGTGCTGCTAC | CAGTGTCCCACTTGGTACCC |
| *L. helveticus* | *pyrG* | TGCAGCACTCACCACTGATT | CCTTTTGGTCCATGCCTTGC |
| *L. plantarum* | *fusA* | ACCATTACCTCTGCTGCCAC | GCCGTCATCGATGTCAGCTA |
| *L. reuteri* | *pyrG* | GAAAGGGAATCGTTGCTGCC | CACCAAGGTAATCCCCACGG |
| *L. rhamnosus GG* | *leuS* | AGCAGCACATAAGTCCGATCT | AAGACCCAGTCACGCAACTT |
| *L. salivarius* | *pyrG* | CGTGACAGATGACGGTACAGA | TGTGATTGTACCTGCACGCA |
| *L. delbrueckii subsp. bulgaricus* | *fusA* | GATCACTTCCGCAGCCACTA | CGATAGGCATTTGAACGGCG |
| *L. zeae* | *fusA* | AACATCATCGATACCCCGGG | CCTTTTGGGCTGCTTCCTTG |
| *Lactococcus lactis* | *bcaT* | CACCTGCTTTGCATTATGGTCA | ACCCCAATCAAAAGTGGACG |
| *S. thermophilus* | *leuS* | CGTGGTGGCTATCCAGTTGT | CCTGTTCCGTAGCTAGCGAG |

a. DNA primer pair sequences (forward and reverse) listed 5’ to 3’ for amplification of respective gene.
