# Supplementary material for: Culture-Independent Metagenomic Surveillance of Commercially Available Probiotics with High-Throughput Next-Generation Sequencing
Source: mSphere. 2016 Mar 30;1(2):e00057-16. doi: 10.1128/mSphere.00057-16 (PMC4894680; doi:10.1128/mSphere.00057-16)
Supplement: TABLE S4 [file sph002162055st4.docx]

**Table S4**

| **K-mer Species ID** | **1.8 billion^b^** | | **2 billion** | | **5 billion** | | **6.5 billion** | |
| --- | --- | --- | --- | --- | --- | --- | --- | --- |
|  | **Signature counts** | **%** | **Signature counts** | **%** | **Signature counts** |  | **Signature counts** | **%** |
| ***Bifidobacterium animalis*** | 670553 | 33.142 | 673925 | 29.094 | 732267 | 27.929 | 745131 | 27.887 |
| ***Bifidobacterium longum*** | 120012 | 8.674 | 118334 | 7.471 | 123147 | 6.869 | 129995 | 7.115 |
| ***Enterococcus faecium*** | **39028** | **1.527** | **233858** | **7.991** | **493744** | **14.906** | **540303** | **16.005** |
| ***Lactobacillus acidophilus*** | 813795 | 39.893 | 933305 | 39.963 | 962558 | 36.413 | 945472 | 35.096 |
| ***Lactobacillus casei group*** | 9726 | 0.471 | 11176 | 0.473 | 10963 | 0.410 | 11143 | 0.409 |
| ***Lactobacillus plantarum*** | 297594 | 11.927 | 312020 | 10.923 | 317288 | 9.813 | 331016 | 10.046 |
| ***Lactobacillus rhamnosus*** | 28871 | 1.270 | 31333 | 1.204 | 32330 | 1.097 | 33305 | 1.109 |
| ***Lactobacillus salivarius*** | 7747 | 0.545 | 8308 | 0.510 | 9296 | 0.505 | 8581 | 0.457 |
| ***Streptococcus thermophilus*** | 13757 | 0.754 | 16233 | 0.777 | 16554 | 0.700 | 15623 | 0.648 |
| ***Lactobacillus zeae*** | 8791 | 0.282 | 9380 | 0.263 | 10395 | 0.258 | 10990 | 0.267 |
| ***Bifidobacterium breve*** | 25731 | 1.370 | 25822 | 1.201 | 24376 | 1.002 | 21507 | 0.867 |
| **Sequencing Run Data** | | | | | | | | |
| **Median seg length R1** | 142 | | 113 | | 98 | | 99 | |
| **Median seg length R2** | 114 | | 104 | | 96 | | 97 | |
| **Total read count** | 2666691 | | 3113448 | | 3466714 | | 3497074 | |
| **No match** | 628618 | | 737258 | | 731581 | | 701812 | |

1. Representing 500mg (50 billion CFU) Product G.
2. Spiked level of *E. faecium* in CFU.
