# Supplementary material for: Culture-Independent Metagenomic Surveillance of Commercially Available Probiotics with High-Throughput Next-Generation Sequencing
Source: mSphere. 2016 Mar 30;1(2):e00057-16. doi: 10.1128/mSphere.00057-16 (PMC4894680; doi:10.1128/mSphere.00057-16)
Supplement: TABLE S5 [file sph002162055st5.docx]

**Table S5**

| **Sample** | **Labeled ingredients** | **Sequencing results^a^** | | | | | **Culture** | **PCR** | **Array** | |
| --- | --- | --- | --- | --- | --- | --- | --- | --- | --- | --- |
| **Product A** |  |  | **Lot 1** | **Lot 2** | **Lot 3** | | **Lot 1** | **Lot 1** | ND^b^ | |
|  |  | Total read count | 2302737 | 2205798 | 28588996 | |  |  |  |  |
|  |  | Unmatched reads | 1038609 | 986147 | 13070723 | |  |  |  |  |
|  |  |  | **% read** | **% read** | **% read** | |  |  |  |  |
|  | *B. infantis* | *B. longum* | 98.68 | 99.62 | 99.6 | | √ | √ |  |  |
|  |  | *B. breve* | 0.23 | 0.24 | 0.24 | |  |  |  |  |
|  | | | | | | | | | | |
| **Product B** |  |  | **Lot 1** | **Lot 2** | **Lot 3** | | **Lot 1** | **Lot 1** | ND | |
|  |  | Total read count | 669101 | 4145674 | 177132139 | |  |  |  |  |
|  |  | Unmatched reads | 196813 | 1384303 | 5320703 | |  |  |  |  |
|  |  |  | **% read** | **% read** | **% read** | |  |  |  |  |
|  | *Lactobacillus rhamnosus GG* | *Lactobacillus rhamnosus* | 99.98 | 99.88 | 99.98 | | √ | √ |  |  |
|  | | | | | | | | | | |
| **Product C** |  |  | **Lot 1** | **Lot 2** | **Lot 3** | | **Lot 1** | **Lot 1** | ND | |
|  |  | Total read count | ND | 1582877 | 7132139 | |  |  |  |  |
|  |  | Unmatched reads |  | 446767 | 2252874 | |  |  |  |  |
|  |  |  | **% read** | **% read** | **% read** | |  |  |  |  |
|  | *Lactobacillus rhamnosus GG* | *Lactobacillus rhamnosus* | ND | 99.81 | 99.98 | | √ | √ |  |  |
|  | | | | | | | | | | |
| **Product D** |  |  | **Lot 1** | **Lot 2** | **Lot 3** | | **Lot 1** | **Lot 1** | ND | |
|  |  | Total read count | 5174806 | 11477017 | 15367316 | |  |  |  |  |
|  |  | Unmatched reads | 2055986 | 4799458 | 5976129 | |  |  |  |  |
|  |  |  | **% read** | **% read** | **% read** | |  |  |  |  |
|  | *L. acidophilus* | *L. acidophilus* | 68.34 | 11.95 | 10.32 | | √ | √ |  |  |
|  | *B. longum* | *B. longum* | 17.58 | 63.32 | 66.2 | | √ | √ |  |  |
|  | *B. bifidum* | *B. bifidum* | 6.98 | 23.09 | 20.81 | | √ | √ |  |  |
|  | *L. plantarum* | *L. plantarum* | 6.65 | **1.42** | 2.09 | | √ | √ |  |  |
|  |  | *B. animalis* | **0.116** |  |  | |  |  |  |  |
|  |  | *L. rhamnosus* |  |  | **0.42** | |  |  |  |  |
|  |  | *L. reuteri* |  |  | **0.12** | |  |  |  |  |
|  | | | | | | | | | | |
| **Product E** |  |  | **Lot 1** | **Lot 2** | **Lot 3** | | **Lot 1** | **Lot 1** | ND | |
|  |  | Total read count | 23772292 | 9757257 | 5735927 | |  |  |  |  |
|  |  | Unmatched reads | 5466487 | 2195635 | 2810360 | |  |  |  |  |
|  |  |  | **% read** | **% read** | **% read** | |  |  |  |  |
|  | *L. acidophilus* | *L. acidophilus* | 48.2 | 35.1 | 30.63 | | √ | √ |  |  |
|  | *B. longum* | *B. longum* | 27.75 | 31.55 | 22.83 | | √ | √ |  |  |
|  | *B. lactis* | *B. animalis subsp. lactis* | 13.85 | 20.22 | 28.45 | | √ | √ |  |  |
|  | *B. breve* | *B. breve* | 4.34 | 6.34 | 3.29 | | √ | √ |  |  |
|  | *L. rhamnosus* | *L. rhamnosus* | 2.82 | 3.48 | 1.56 | | √ | √ |  |  |
|  | *L. plantarum* | *L. plantarum* | **1.24** | **1.39** | 8.16 | | √ | √ |  |  |
|  | *L. salivarius* | *L. salivarius* | **0.96** | **1.004** | **0.59** | |  |  |  |  |
|  |  | *L. zeae* | **0.74** | **0.71** | **0.67** | |  |  |  |  |
|  |  | *S. thermophilus* |  |  | **0.49** | |  |  |  |  |
|  |  | *Lactococcus lactis subsp. lactis* |  |  | **0.199** | |  |  |  |  |
|  |  | *Lactobacillus brevis* |  |  | **0.626** | |  |  |  |  |
|  |  | *Lactobacillus gasseri* |  |  | **0.213** | |  |  |  |  |
|  | *L. casei* | *Lactobacillus casei group* |  |  | 1.97 | |  |  |  |  |
|  | | | | | | | | | | |
| **Product F** |  |  | **Lot 1** | **Lot 2** | **Lot 3** | | **Lot 1** | **Lot 1** | ND | |
|  |  | Total read count | 9536788 | 586410 | 2775322 | |  |  |  |  |
|  |  | Unmatched reads | 3229632 | 243208 | 1229747 | |  |  |  |  |
|  |  |  | **% read** | **% read** | **% read** | |  |  |  |  |
|  | *L. acidophilus* | *L. acidophilus* | 41.21 | 41.21 | 26.78 | | √ | √ |  |  |
|  | *B. longum* | *B. longum* | 27.79 | 18.01 | 31.75 | | √ | √ |  |  |
|  | *L. rhamnosus* | *L. rhamnosus* | 11.08 | 17.18 | 15.01 | | √ | √ |  |  |
|  | *B. lactis* | *B. animalis subsp. lactis* | 7.91 | 9.85 | 10.42 | | √ | √ |  |  |
|  | *L. casei* | *L. casei* | 7.78 | 7.06 | 7.5 | | √ | √ |  |  |
|  | *L. plantarum* | *L. plantarum* | 3.59 | 6.32 | 7.65 | | √ | √ |  |  |
|  |  | *Lactobacillus helveticus* | **0.25** |  |  | |  |  |  |  |
|  |  | *B. bifidum* | **0.14** | **0.14** | **0.18** | |  |  |  |  |
|  |  | *L. reuteri* |  |  | **0.49** | |  |  |  |  |
|  | | | | | | | | | | |
| **Product G** |  |  | **Lot 1** | **Lot 2** | **Lot 3** | | **Lot 1** | **Lot 1** | √ | |
|  |  | Total read count | 20250068 | 5231310 | 5111203 | |  |  |  |  |
|  |  | Unmatched reads | 6737627 | 1167931 | 1554081 | |  |  |  |  |
|  |  |  | **% read** | **% read** | **% read** | |  |  |  |  |
|  | *L. rhamnosus* | *L. rhamnosus* | 37.53 | 2.24 | 1.5 | | √ | √ |  |  |
|  | *L. acidophilus* | *L. acidophilus* | 19.8 | 33 | 47.1 | | √ | √ |  |  |
|  | *L. casei and  L. paracasei* | *L. casei group* | 18.24 | **0.62** | **0.56** | | √ | √ |  |  |
|  | *L. reuteri*^c^ | *L. reuteri* | 8.61 | **0.32** |  | |  |  |  |  |
|  |  | *E.faecium* | 6.22 |  |  | | √ | √ |  |  |
|  | *B. lactis* | *B. animalis subsp. lactis* | 6.12 | 31.47 | 33.58 | | √ | √ |  |  |
|  | *L. plantarum* | *L. plantarum* | 1.54 | 19.24 | 9.44 | | √ | √ |  |  |
|  | *L. salivarius* | *L. salivarius* | **0.92** | **0.33** | **0.66** | |  |  |  |  |
|  |  | *Clostridium perfringens* | **0.28** |  |  | |  |  |  |  |
|  |  | *Pediococcus acidilacti* | **0.26** |  |  | |  |  |  |  |
|  |  | *L. zeae* | **0.22** | **0.499** | **0.28** | |  |  |  |  |
|  | *S. thermophilus* | *S. thermophilus* | **0.14** | **1.003** | **0.45** | |  |  |  |  |
|  | *B. longum* | *B. longum* |  | 8.7 | 4.67 | | √ | √ |  |  |
|  | *B. breve*^d^ | *B. breve* |  | 2.37 | 1.59 | |  |  |  |  |
|  | | | | | | | | | | |
| **Product H** |  |  | **Lot 1** | **Lot 2** | **Lot 3** | **Lot 1** | | **Lot 1** | | √ |
|  |  | Total read count | 7280084 | 3882943 | 16666035 |  | |  | |  |
|  |  | Unmatched reads | 4290940 | 1170389 | 5258515 |  | |  | |  |
|  |  |  | **% read** | **% read** | **% read** |  | |  | |  |
|  | *B. bifidum* | *B. bifidum* | 58.85 | 43.4 | 48.1 | √ | | √ | |  |
|  | *B. longum* | *B. longum* | 23.37 | 25.56 | 25.63 | √ | | √ | |  |
|  | *B. breve* | *B. breve* | 11.6 | 19.15 | 13.2 | √ | | √ | |  |
|  | *L. acidophilus* |  |  |  |  |  | |  | |  |
|  |  | *L. helveticus* | 3.17 | 3.65 | 5.38 | √ | | √ | |  |
|  | *L. rhamnosus* | *L. rhamnosus* | 2.96 | 8.18 | 7.6 | √ | | √ | |  |
|  | | | | | | | | | | |
| **Product I** |  |  |  |  |  | |  |  | | ND |
|  |  |  | **Lot 1** | **Lot 2** | **Lot 3** | | **Lot 1, 2, 3** | **Lot 1, 2, 3** | |  |
|  |  | Total read count | 9816271 | 4146281 | 7456108 | |  |  | |  |
|  |  | Unmatched reads | 3970681 | 1820893 | 3456952 | |  |  | |  |
|  |  |  | **% read** | **% read** | **% read** | |  |  | |  |
|  | *L. rhamnosus* | *L. rhamnosus* | 33.46 | 22.69 | 21.45 | | √ | √ | |  |
|  | *L. reuteri* | *L. reuteri* | 66.28 | 76.81 | 77.51 | | √ | √ | |  |
|  |  | *B. bifidum* |  |  | **0.108** | |  |  | |  |
|  |  | *B. longum* |  | **0.114** | **0.734** | |  |  | |  |
|  |  | *L. acidophilus* |  | **0.177** |  | |  |  | |  |
|  | | | | | | | | | | |
| **Product J** |  |  | **Lot 1** | **Lot 2** | **Lot 3** | | **Lot 1** | **Lot 1** | | ND |
|  |  | Total read count | 24949251 | 23652636 | 2806661 | |  |  | |  |
|  |  | Unmatched reads | 6807236 | 6916032 | 769867 | |  |  | |  |
|  |  |  | **% read** | **% read** | **% read** | |  |  | |  |
|  | *L. acidophilus* | *Lactobacillus acidophilus* | 53.68 | 42.46 | 52.56 | | √ | √ | |  |
|  | *B. lactis HN019* | *Bifidobacterium animalis subsp. lactis* | 17.95 | 24.34 | 15.103 | | √ | √ | |  |
|  | *L. plantarum* | *Lactobacillus plantarum* | 13.36 | 16.28 | 12.623 | | √ | √ | |  |
|  | *B. longum* | *Bifidobacterium longum* | 5.68 | 6.79 | 9.35 | | √ | √ | |  |
|  | *L. paracasei and L. casei* | *Lactobacillus casei group* | 2.752 | 2.45 | 2.833 | | √ | √ | |  |
|  | *S. thermophilus* | *Streptococcus thermophilus* | **1.03** | **1.36** | **0.875** | | √ | √ | |  |
|  | *L. lactis* | *Lactococcus lactis subsp. lactis* | **0.961** | **0.91** | **1.06** | |  |  | |  |
|  |  | *Lactobacillus zeae* | **0.913** | **0.96** | **0.725** | |  |  | |  |
|  | *L. salivarius* | *Lactobacillus salivarius* | **0.891** | **0.92** | **1.003** | |  |  | |  |
|  | *L. brevis* | *Lactobacillus brevis* | **0.804** | **0.75** | **0.83** | |  |  | |  |
|  | *L. gasseri* | *Lactobacillus gasseri* | **0.784** | **0.71** | **0.802** | |  |  | |  |
|  | *B. breve* | *Bifidobacterium breve* | **0.327** | **1.03** | **0.92** | | √ | √ | |  |
|  | *L. rhamnosus* | *Lactobacillus rhamnosus* | **0.234** | **0.26** | **0.797** | | √ | √ | |  |
|  |  | *Lactococcus lactis subsp. cremoris* | **0.224** | **0.22** | **0.175** | |  |  | |  |
|  | *L. bulgaricus* | *Lactobacillus delbrueckii subsp. bulgaricus* | **0.191** | **0.16** |  | |  |  | |  |
|  |  | *Lactobacillus helveticus* |  | **0.107** |  | |  |  | |  |
|  | *B. bifidum* |  |  |  |  | |  |  | |  |
|  | *B. lactis (infantis)* |  |  |  |  | |  |  | |  |

1. Percentages **in bold** are below 1.5%, the cut-off for a presumptive present call.
2. ND, not determined.
3. Listed only on lot 1 label.
4. In lots 2 and 3, *L. reuteri* was replaced with *B. breve*.
