# Supplementary material for: Culture-Independent Metagenomic Surveillance of Commercially Available Probiotics with High-Throughput Next-Generation Sequencing
Source: mSphere. 2016 Mar 30;1(2):e00057-16. doi: 10.1128/mSphere.00057-16 (PMC4894680; doi:10.1128/mSphere.00057-16)
Supplement: TABLE S6 [file sph002162055st6.docx]

**Table S6**

| **Organism** | **Medium** | **Conditions^a^** | **Colony Description** |
| --- | --- | --- | --- |
| *S. thermophilus, L. delbruckeii ssp. Bulgaricus* | MRS | 37 C , 3 Days, Anaerobic | *S. thermophilus*= circular, opalescent colonies with well-defined border, *L. delb. Ssp. bulgaricus*= rounded colonies |
|  | MRS-Lactose | 37 C , 3 Days, Aerobic | *S. thermophilus*= circular, opalescent colonies with well-defined border, *L. delb. ssp. bulgaricus*= bigger, irregular translucent colonies with non-defined borders |
|  | MRS-Glucose |  |  |
| *L. acidophilus, L. paracasei, L. casei* | MRS-Raffinose | 37 C , 2 days, Anaerobic | *L. casei*= circular, milky white colonies, defined. *L. paracasei*= brown/off white colonies, defined circular, *L. acidophilus*= irregular edges, brown/off white colonies |
|  | MRS-Lactose |  |  |
|  | MRS-Glucose |  |  |
| *Bifiobacterium sp.* | Bifidobacterium agar | 37 C, 2 days, Anaerobic | *Bifidobacterium longum*= pink colonies with dark centre and tiny white edges, *B. breve*= tiny pink colonies |
| *L. acidophilus, Bifidobacterium* | MRS-Maltose | 37 C, 3 days, Anaerobic | *Bifidobacterium*= growth is inhibited, *L. acidophilus*= irregular edges, brown/off white colonies |
|  | MRS-Aarabinose |  | *Bifidobacterium bifidum*, *B. infantis*, *B. breve*= growth is inhibited, *B. longum* and *B. pseudolongum*= tiny white dot colonies, *L. acidophilus*= white colonies with irregular edges |
|  | MRS-Glucose |  | *Bifidobacterium* = growth inhibited, *L. acidophilus*= rounded colonies, white and defined edges |
|  | RCM |  | *L. acidophilus*= rounded colonies, white and defined edges |
| *L. casei, L. paracasei, L. salivarius, L. reuteri* | MRS-Raffinose | 37 C, 3 days, Anaerobic | Impossible to differentiate between *L. salivarius*, *L. reuteri*, *L. casei* and *L. paracasei*. Rounded edge, off white, pinpoint colonies |
|  | MRS-Maltose |  |  |
|  | MRS-Lactose |  |  |
|  | MRS-Glucose |  |  |
| *E. faecium* | Enterococcus media | 37 C, O/N, Aerobic | *E. faecium*= rounded, off white, tiny colonies |

1. Temperature, time, and atmosphere.
