# Supplementary material for: Culture-Independent Metagenomic Surveillance of Commercially Available Probiotics with High-Throughput Next-Generation Sequencing
Source: mSphere. 2016 Mar 30;1(2):e00057-16. doi: 10.1128/mSphere.00057-16 (PMC4894680; doi:10.1128/mSphere.00057-16)
Supplement: TABLE S7 [file sph002162055st7.docx]

**Table S7**

| **Sample** | **Ingredients** | **Medium^a^** | | | | | | | | | | | | | | |
| --- | --- | --- | --- | --- | --- | --- | --- | --- | --- | --- | --- | --- | --- | --- | --- | --- |
|  |  |  | | | | | | | | | | | | | | |
|  |  | **MRS** | **MRS-Lactose** | **MRS-Raffinose** | **MRS-Sucrose** | **MRS-Lactulose** | **Bifidobacterium** | **M-17** | **M-17 Lactose** | **CSM** | **RCM** | **PDA** | **LB** | **TSA** | **MacConkey** | **Rogosa** |
|  |  |  |  |  |  |  |  |  |  |  |  |  |  |  |  |  |
| Product A | Bifantis (*Bifidobacterium infantis* 35624) contains 1 x 10^9^ CFU (1 billion) (4 mg) when manufactured and provides an effective level of bacteria (1 x 10^7^) until at least the "best by" date | x | x | x | x | x | √ | x | x | x | x | x | x | x | x | x |
| Product B | 10 billion cells: *Lactobacillus GG,* 10 billion cells: Inulin (chicory root extract) 200 mg | √ | √ | √ | √ | √ | x | √ | √ | x | x | x | x | x | x | x |
| Product C | 5 billion cells: *Lactobacillus GG* | √ | √ | √ | √ | √ | x | √ | √ | x | x | x | x | x | x | x |
| Product D | 4 billion cells: *L. acidophilus*, *B. Bifidum*, *B. Longum*, *L. Plantarum* OM | √ | √ | √ | √ | √ | √ | √ | √ | x | x | x | x | x | x | x |
| Product E | *Lactobacillus acidophilus* (La-14) 12 Billion CFU's, *Bifidobacterium lactis* (BI-04) 12 Billion CFU's, *Lactobacillus casei* (Lc-11) 1 Billion CFU's, *Bifidobacterium breve* (Bb-03) 1 Billion CFU's, *Lactobacillus salivarius* (Ls-33) 1 Billion CFU's, *Lactobacillus plantarum* (Lp-115) 1 Billion CFU's, *Bifidobacterium longum* (Bl-05) 1 Billion CFU's,  *Lactobacillus rhamnosus* (Lr-32) 1 Billion CFU's | √ | √ | √ | √ | √ | x | √ | √ | x | x | x | x | x | x | x |
| Product F | 3.4 billion CFU's: *B. longum* (BB536), *L. acidophilus* (La-14), *B. lactis (BI-04),* *L. rhamnosus* (R0011), *L. casei* (R0215), *L. plantarum* (R1012) | √ | √ | √ | √ | √ | √ | √ | √ | x | x | x | x | x | x | x |
| Product G | Proprietary Blend of 10 Strains of Probiotic Bacteria 50 Billion Organisms: *Lactobacillus paracasei, Bifidobacterium lactis, Lactobacillus plantarum, Lactobacillus acidophilus, Bifidobacterium longum, Lactobacillus rhamnosus , Streptococcus thermophilus, Lactobacillus casei, Lactobacillus salivarius, Lactobacillus reuteri (*Lot 1)*,*  *Bifidobacterium breve* (Lots 2 & 3) | √ | √ | √ | √ | √ | √ | √ | √ | x | x | x | x | x | x | x |
| Product H | 100 Billion CFU's: *Bifidobacterium breve*-129, *Bifidobacterium longum*-135, *Bifidobacterium bifidum*-132, *Lactobacillus acidophilus-*122, *Lactobacillus rhamnosus*-111 | √ | √ | √ | √ | √ | √ | √ | √ | x | x | x | x | x | x | x |
| Product I | 2.5 Billion cfu: *L. rhamnosus* GR-1, 2.5 Billion cfu: *L- reuteri* RC-14 | √ | √ | √ | √ | √ | x | √ | √ | x | x | x | x | x | x | x |
| Product J | 32 billion cells: *Bifidobacterium bifidum*, *Bifidobacterium breve,* *Bifidobacterium lactis (infantis)*, *Bifidobacterium lactis HN019, Bifidobacterium longum, Lactobacillus acidophilus, Lactobacillus brevis, Lactobacillus bulgaricus, Lactobacillus casei*, *Lactobacillus gasseri*, *Lactobacillus paracasei*, *Lactobacillus plantarum*, *Lactobacillus rhamnosus*, *Lactobacillus salivarius*, *Lactococcus lactis*, *Streptococcus thermophilus* | √ | √ | √ | √ | √ | √ | √ | √ | x | x | x | x | x | x | x |

a. Probiotics were plated on various strain-specific media and growth was recorded; “x” indicates no growth, “√” indicates presence of colonies.
